# Pharmacological advances of the chlorogenic acids family: current insights and future research directions

**DOI:** 10.3389/fphar.2025.1613048

**Published:** 2025-10-10

**Authors:** Qin Yuan, Can Liu, Zian Zhang, Fan Chen, Qiang Xiao, Liangqi Zhang, Xue Pan, Fuyuan He, Meifeng Xiao

**Affiliations:** ^1^ College of Pharmacy, Hunan University of Chinese Medicine, Changsha, China; ^2^ Hunan Provincial Key Laboratory of Drugability and Preparation Modification of Traditional Chinese Medicine, Changsha, China

**Keywords:** chlorogenic acids, pharmacological effects, mechanisms of action, phenolic acids, traditional Chinese medicine

## Abstract

Phenolic acids are considered an important source for developing natural drugs due to their extensive biological activities. The chlorogenic acids (CGAs) family is the most widely distributed botanical drug in the phenolic acid group and is also commonly found in various traditional Chinese medicine (TCM) extracts. The diverse structural variations of naturally occurring chlorogenic acids result in distinct functions and mechanisms. Recent studies have demonstrated that chlorogenic acid can reduce Aβ plaques in Alzheimer’s disease model mice by 37%, indicating its neuroprotective potential. Similarly, CGAs offer protection to the cardiovascular system, gastrointestinal tract, kidneys, and liver, while additionally preventing metabolic syndrome and displaying anticancer and antimicrobial capabilities. The key signaling pathways and factors involved in these effects include PI3K/AKT, NF-κB, JNK, NLRP3, and Keap1/Nrf2. This review, for the first time, provides a comparative analysis of six typical CGAs, systematically reviewing their specific distribution characteristics in traditional Chinese medicinal metabolites, biosynthetic pathways, biological targets, and pharmacological activities. This review provides a reference for the research and rational development and utilization of CGAs.

## 1 Introduction

As global public health consciousness has significantly increased (Leonardi et al., 2024) and traditional Chinese medicine (TCM) has gained increasing recognition in the international medical community (Lyu et al., 2021; Gao et al., 2022), the market demand for TCM has continued to grow, thereby driving the rapid development of the TCM industry (Xiang et al., 2022). Under this background, CGAs, as an important group of bioactive substances, play a key role. Notably, CGAs are among the most significant polyphenols in the daily diet, being widely distributed in coffee, fruits (such as apples, pears, and berries), and vegetables (such as potatoes and artichokes). This widespread presence underpins their extensive physiological effects. Driven by mass spectrometry technology, CGAs can be divided into two major categories, namely monochlorogenic acids and dichlorogenic acids, based on the binding sites and numbers of caffeic acid and quinic acid. Their antioxidant and anti-inflammatory mechanisms have been proven to have multi-target regulatory effects on nervous system diseases. With the international natural pharmaceutical market size exceeding 50 billion US dollars, the application fields of CGAs are continuously expanding. Their clinical value is increasingly widely recognized by the academic community and has gradually become a research hotspot.

**FIGURE 1:**
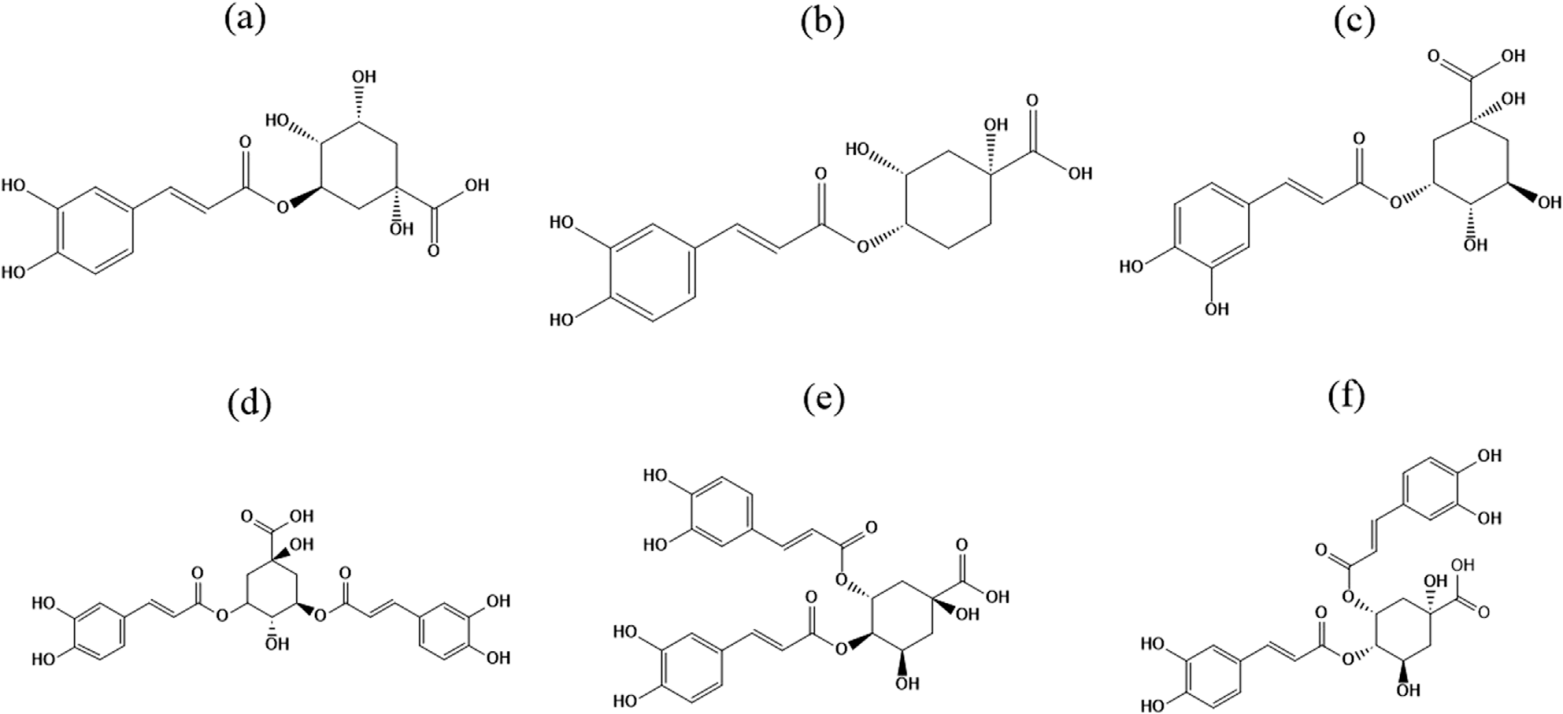
Chemical structures of six chlorogenic acids. **(a)** Chlorogenic acid (5-CQA); **(b)** Cryptochlorogenic acid (4-CQA); **(c)** Neochlorogenic acid (3-CQA); **(d)** Isochlorogenic acid A (3,5-di-CQA); **(e)** Isochlorogenic acid B (4,5-di-CQA); **(f)** Isochlorogenic acid C (3,4-di-CQA).

CGAs are natural phenolic acid compounds that result from the esterification of caffeic acid and quinic acid. Mono-caffeoyl quinic acids and di-caffeoyl quinic acids are the two main groups into which CGAs can be separated based on the binding sites and quantities of caffeic acid and quinic acid moieties. Chlorogenic acid (CGA), neochlorogenic acid (NCGA), and cryptochlorogenic acid (CCGA) are the most common mono-caffeoyl quinic acids; isochlorogenic acid A (ICGA-A), isochlorogenic acid B (ICGA-B), and isochlorogenic acid C (ICGA-C) are the most common di-caffeoyl quinic acids. According to IUPAC numbering conventions, CGA is designated as 5-O-caffeoyl quinic acid (5-CQA), however, other researchers refer to it as 3-CQA. Figure 1 delineates the chemical structures of these six CGAs. Research indicates that CGAs demonstrate a diverse array of biological activities and pharmacological effects, encompassing anti-inflammatory, antioxidant, antibacterial, neuroprotective, cardioprotective, hepatorenal protective, metabolic syndrome therapy, and anticancer properties (Yu et al., 2022). These diverse pharmacological effects offer broad prospects for their application in the medical field. However, research on the distribution characteristics and content differences of CGAs in traditional Chinese medicinal materials remains insufficient, and comparative studies on the pharmacological activities and mechanisms of action of different CGAs are also relatively limited, highlighting important directions for future research.

This review comprehensively searched for literature in databases such as PubMed, ScienceDirect, Google Scholar, and Web of Science to identify studies related to the chlorogenic acid family. The following topics or keywords were searched: “CGA,” “chlorogenic acid,” “neochlorogenic acid,” “cryptochlorogenic acid,” “Isochlorogenic acid A”, “Isochlorogenic acid B,” “Isochlorogenic acid C,” “chlorogenic acid derivatives,” The search period was from 2010 to 2025, and the core literature of this review mainly focused on the period from 2020 to 2025. The inclusion criteria included English original articles and English review articles that discussed the sources of the chlorogenic acid family, biosynthetic pathways, biological targets, and pharmacological activities. In addition, when summarizing the evidence system of this research field, we conducted a methodological review of the model applicability of the included studies through a systematic literature quality assessment framework. We strictly excluded purely computational simulation studies that lacked experimental validation, focusing on experimental studies with multidimensional validation characteristics. Most of the literature retrieved in this article used *in vivo* and *in vitro* models for synergistic experimental validation, effectively ensuring the integrity of the evidence chain.

## 2 Discovery and primary plant sources of CGAs

In 1897, Osborne and Campbell discovered that sunflower seeds contained a substance named Helianthotannic acid, which was the primary cause of protein browning in sunflower seeds (Osborne and Campbell, 1897). In 1909, Gorter devised a quick screening technique for CGA and successfully recovered high-purity CGA crystals from coffee beans (Dai et al., 2024b). Between 1950 and 1964, three additional monochlorogenic acid isomers, namely neochlorogenic acid (NCGA) (Barnes et al., 1950), pseudochlorogenic acid (Hulme, 1953), and cryptochlorogenic acid (CCGA) (Kremr et al., 2016) were successively identified.

CGAs represent a significant category of phenolic botanical drugs, commonly found in higher dicotyledonous plants and ferns. Their distribution characteristics indicate that these botanical drugs are particularly abundant in the Lonicera genus of the Caprifoliaceae family and the Artemisia genus of the Asteraceae family. In medicinal plant resources*, Lonicera japonica* Thunb., and *Eucommia ulmoides* Oliv. have been identified as species with high accumulation of CGA. Additionally, CGA exhibits high bioavailability in various dietary sources, with typical representatives including coffee beans, green tea, and strawberries. This distribution pattern suggests that CGA plays a significant ecological and physiological role among plant secondary metabolites. In addition, this paper utilized the search terms “chlorogenic acid,” “traditional Chinese medicine” and “plant” to search the PubMed, Web of Science, CNKI, VIP, and WanFang databases. The inclusion criteria were set to experimental research articles that identified CGA, NCGA, CCGA, and ICGA in the chemical composition of traditional Chinese medicine. Review articles, duplicates, and articles lacking quantitative data were excluded. After screening, a total of 197 chlorogenic acid-related pharmaceutical articles were selected. The traditional Chinese medicine metabolites that are rich in CGAs are summarized and presented in Table 1.

**TABLE 1:** Distribution of six chlorogenic acids in traditional Chinese medicinal plants.

Name	Plant source
CGA (5-CQA)	*Lonicera macranthoides* Hand.-Mazz., *Dracocephalum moldavica* L., *Lonicera japonica* Thunb., *Eucommia ulmoides* Oliv., *Juglans regia* flower, *Chrysanthemum × morifolium* (Ramat.) Hemsl., *Lonicera hypoglauca* Miq., *Saussurea involucrata* (Kar. & Kir.) Sch.Bip., *Lonicera japonica* Thunb., *Beckmannia syzigachne* (Steud.) Fernald, *Pyrrosia sheareri* (Baker) Ching, *Pyrrosia petiolosa* (Christ) Ching, *Xanthium strumarium* L., *Prunus mume* (Siebold) Siebold & Zucc., *Morus alba* L., *Artemisia capillaris* Thunb., *Artemisia vulgaris* L., *Mussaenda pubescens* Dryand., *Stevia rebaudiana* (Bertoni) Bertoni, *Helianthus annuus* L., *Aster tataricus* L. f.,*Artemisia tilesii* Ledeb., *Cynara cardunculus* subsp. *cardunculus*, *Taraxacum scariosum* (Tausch) Kirschner & Štěpánek, Radix Bupleuri, *Blumea riparia* DC., *Cuscuta chinensis* Lam.,*Phellodendron chinense* C.K.Schneid., *Imperata cylindrica* (L.) Raeusch., *Ipomoea pes-caprae* (L.) R. Br., *Ainsliaea fragrans* Champ. ex Benth., *Tetradium ruticarpum* (A.Juss.) T.G.Hartley., *Erycibe obtusifolia* Benth., *Lepisorus carnosus* (Hook.) C.F.Zhao, R.Wei & X.C.Zhang, *Gardenia jasminoides* J.Ellis, *Coriandrum sativum* L., *Lysimachia christinae* Hance, *Pyrrosia lingua* (Thunb.) Farw., *Acanthopanax senticosus* (Rupr. et Maxim.) Harms, *Cephalanoplos segetum* Bge.Kitam., *Arctium lappa* L., *Codonopsis pilosula* (Franch.) Nannf., *Taraxacum mongolicum* Hand.-Mazz., *Viola philippica* Cav., *Stemona japonica* (Blume) Miq., *Sargentodoxa cuneata* (Oliv.) Rehder & E.H.Wilson, *Strychnos nux-vomica* L., *Tussilago farfara* Linn., *Ilex chinensis* Sims, *Sambucus javanica* subsp. *chinensis* (Lindl.) Fukuoka, *Lycium barbarum* leaf, *Galium verum* L., *Centella asiatica* (L.) Urb., *Solanum virginianum* L., *Schefflera venulosa* (Wight et Arn.) Harms, *Notopterygium incisum* Ting ex H. T. Chang, *Gongronemopsis tenacissima* (Roxb.) S.Reuss, Liede & Meve, *Echinacea purpurea*, *Centipeda minima* (L.) A.Braun & Asch., *Conioselinum anthriscoides* ‘Chuanxiong’, *Ipomoea nil* (L.) Roth, *Platycodon grandiflorus* (Jacq.) A. DC., *Pinellia ternata* (Thunb.) Makino., *Saururus chinensis* (Lour.) Baill., *Cyclocodon lancifolius* (Roxb.) Kurz, *Saxifraga stolonifera* Curtis, *Angelica gigas* Nakai, *Glycine max* (L.) Merr., *Zanthoxylum bungeanum* Maxim., *Lepidium meyenii* Walp., *Periploca forrestii* Schltr., *Bistorta officinalis* Delarbre, *Coreopsis tinctoria* Nutt., *Gynura crepidioides* Benth., *Arctium lappa* L., *Phanera championii* Benth., *Saussurea hieracioides* Hook. f., *Houttuynia cordata* Thunb., *Saxifraga stolonifera* Curt., Herba Ardisiae Japonicae, *Chrysanthemum × morifolium* (Ramat.) Hemsl., *Forsythia suspensa* (Thunb.) Vahl, *Pinellia ternata* (Thunb.) Makino, *Prunella vulgaris* L., *Dipsacus japonicus* Miq., *Isodon rubescens* (Hemsl.) H. Hara, *Eleutherococcus giraldii* (Harms) Nakai, *Valeriana officinalis* Linn., *Ephedra sinica* Stapf, *Erigeron breviscapus* (Vaniot) Hand.-Mazz., *Imperata cylindrica* (L.) Raeusch., *Alnus nepalensis* D.Don, *Vatica mangachapoi* Blanco, *Helianthus annuus* Linn., *Ilex kaushue* S.Y.Hu, *Artemisia annua* Linn., *Eleutherococcus nodiflorus* (Dunn) S.Y.Hu, *Viola hamiltoniana* D.Don, *Hydrangea macrophylla* (Thunb.) Ser., *Lycium ruthenicum* Murray, *Lycium chinense* Mill., *Helianthus annuus* L., *Helianthus tuberosus* L., *Angelica sinensis* (Oliv.) Diels
NCGA (3-CQA)	*Lonicera japonica* Thunb., *Juglans regia* flower, Chrysanthemi Flos, *Lonicera hypoglauca* Miq., *Pyrrosia petiolosa* (Christ) Ching, *Prunus mume* (Siebold) Siebold & Zucc., *Morus alba* L., *Artemisia capillaris* Thunb., *Artemisia vulgaris* L., *Mussaenda pubescens* Dryand., *Stevia rebaudiana* (Bertoni) Bertoni, *Cynara cardunculus* subsp. *cardunculus*, *Cuscuta chinensis* Lam., *Ipomoea pes-caprae* (L.) R. Br., *Ainsliaea fragrans* Champ. ex Benth., *Tetradium ruticarpum* (A.Juss.) T.G.Hartley, *Erycibe obtusifolia* Benth, *Xanthium strumarium* L., *Eleutherococcus senticosus* (Rupr. & Maxim.) Maxim., *Cephalanoplos segetum* Bge. Kitam., *Arctium lappa* L., *Taraxacum mongolicum* Hand.-Mazz., *Stemona japonica* (Blume) Miq., *Sargentodoxa cuneata* (Oliv.) Rehder & E.H.Wilson, *Tussilago farfara* L., *Lycium barbarum* leaf, *Notopterygium incisum* Ting ex H. T., *Conioselinum anthriscoides* ‘Chuanxiong’, *Ipomoea nil* (L.) Roth, *Periploca forrestii* Schltr., *Houttuynia cordata* Thunb., *Valeriana officinalis* Linn., *Ephedra sinica* Stapf, *Imperata cylindrica* (L.) Raeusch., *Vatica mangachapoi* Blanco, *Artemisia annua* Linn., *Acanthopanax sessiliflorus* (Rupr. et Maxim.) Seem., *Hydrangea macrophylla* (Thunb.) Ser., *Imperata cylindrica* (L.) Raeusch., *Lycium ruthenicum* Murray, *Zanthoxylum austrosinense* C.C.Huang
CCGA (4-CQA)	*Lonicera macranthoides* Hand.-Mazz., *Lonicera japonica* Thunb., *Juglans regia* flower, *Chrysanthemum × morifolium* (Ramat.) Hemsl., *Lonicera hypoglauca* Miq., *Lonicera japonica* Thunb., *Pyrrosia petiolosa* (Christ) Ching, *Prunus mume* (Siebold) Siebold & Zucc., *Morus alba* Linn., *Artemisia capillaris* Thunb., *Artemisia vulgaris* L., *Mussaenda pubescens* Dryand., *Stevia rebaudiana* (Bertoni) Bertoni, *Cynara cardunculus* subsp. *cardunculus*, *Cuscuta chinensis* Lam., *Ipomoea pes-caprae* (L.) R. Br., *Ainsliaea fragrans* Champ. ex Benth., *Evodia rutaecarpa* (Juss.) Benth., *Xanthium strumarium* L., *Eleutherococcus senticosus* (Rupr. & Maxim.) Maxim., *Cephalanoplos segetum* Bge. Kitam., *Arctium lappa* L., *Stemona japonica* (Blume) Miq., *Tussilago farfara* L., *Lycium barbarum* leaf, *Centipeda minima* (L.) A.Braun & Asch., *Conioselinum anthriscoides* ‘Chuanxiong’, *Ipomoea nil* (L.) Roth, *Echinacea purpurea* (L.) Moench, *Periploca forrestii* Schltr., *Arctium lappa* L., *Houttuynia cordata* Thunb., *Imperata cylindrica* (L.) Raeusch., *Vatica mangachapoi* Blanco, *Artemisia annua* L., *Eleutherococcus nodiflorus* (Dunn) S.Y.Hu, *Lycium ruthenicum* Murray
ICGA-A (3,5-CQA)	*Lonicera japonica* Thunb., Chrysanthemi Flos, *Lonicera hypoglauca* Miq., *Lonicera japonica* Thunb., *Beckmannia syzigachne* (Steud.) Fernald, *Xanthium strumarium* L., *Artemisia capillaris* Thunb., *Artemisia vulgaris* L., *Mussaenda pubescens* Dryand., *Stevia rebaudiana* (Bertoni) Bertoni, *Cynara cardunculus* subsp. *cardunculus*, *Bupleurum chinense* DC., *Blumea riparia* DC., *Artemisia annua* L., *Cuscuta chinensis* Lam., *Ipomoea pes-caprae* (L.) R. Br., *Jasminum elongatum* (P.J.Bergius) Willd., *Ainsliaea fragrans* Champ. ex Benth., *Erycibe obtusifolia* Benth., *Arctium lappa* L., *Taraxacum mongolicum* Hand.-Mazz., *Laggera crispata* (Vahl) Hepper & J.R.I.Wood, *Tussilago farfara* L., *Centella asiatica* (L.) Urb., *Centipeda minima* (L.) A.Braun & Asch., *Pharbitis nil* Choisy, *Echinacea purpurea* (L.) Moench, *Angelica sinensis* (Oliv.) Diels, *Bistorta officinalis* Delarbre, *Chrysanthemum × morifolium* (Ramat.) Hemsl., *Arctium lappa* L., *Houttuynia cordata* Thunb., *Periploca forrestii* Schltr., *Dipsacus japonicus* Miq., *Valeriana officinalis* L., *Erigeron breviscapus* (Vaniot) Hand.-Mazz., *Ilex kaushue* S.Y.Hu, *Eleutherococcus nodiflorus* (Dunn) S.Y.Hu, *Lycium ruthenicum* Murray, *Helianthus tuberosus* L.
ICGA-B (4,5-CQA)	*Lonicera japonica* Thunb., *Chrysanthemum × morifolium* (Ramat.) Hemsl., *Lonicera hypoglauca* Miq., *Achillea millefolium* L., *Artemisia capillaris* Thunb., Folium Artemisiae Argyi, *Mussaenda pubescens* Dryand., *Stevia rebaudiana* (Bertoni) Bertoni, *Bupleurum chinense* DC., *Blumea riparia* DC., *Artemisia annua* L., *Cuscuta chinensis* Lam., *Ipomoea pes-caprae* (L.) R. Br., *Jasminum elongatum* (P.J.Bergius) Willd., *Ainsliaea fragrans* Champ. ex Benth., *Erycibe obtusifolia* Benth., *Xanthium strumarium* L., *Taraxacum mongolicum* Hand.-Mazz., *Laggera alata* (D.Don) Sch.Bip. ex Oliv., *Tussilago farfara* L., *Centipeda minima* (L.) A.Braun & Asch., *Ipomoea nil* (L.) Roth, *Echinacea purpurea* (L.) Moench, *Periploca forrestii* Schltr., *Arctium lappa* L., *Saussurea hieracioides* Hook. f., *Dipsacus japonicus* Miq., *Valeriana officinalis* L., *Erigeron breviscapus* (Vaniot) Hand.-Mazz., *Artemisia annua* L., *Acanthopanax sessiliflorus* (Rupr. et Maxim.) Seem., *Lycium ruthenicum* Murray, *Helianthus tuberosus* L.
ICGA-C (3,4-CQA)	*Lonicera japonica* Thunb., *Eucommia ulmoides* Oliv., *Chrysanthemum × morifolium* (Ramat.) Hemsl., *Lonicera hypoglauca* Miq., *Lonicera japonica* Thunb., *Achillea millefolium* L., *Pyrrosia petiolosa* (Christ) Ching, *Xanthium strumarium* L., *Morus alba* L., *Artemisia capillaris* Thunb., *Artemisia vulgaris* L., *Mussaenda pubescens* Dryand., *Stevia rebaudiana* (Bertoni) Bertoni, *Cynara cardunculus* subsp. *cardunculus*, *Blumea riparia* DC., *Artemisia annua* L., *Cuscuta chinensis* Lam., *Ipomoea pes-caprae* (L.) R.Br., *Ainsliaea fragrans* Champ. ex Benth., *Erycibe obtusifolia* Benth., *Taraxacum mongolicum* Hand.-Mazz., *Laggera alata* (D.Don) Sch.Bip. ex Oliv., *Tussilago farfara* L., *Schefflera venulosa* (Wight et Arn.) Harms, *Centipeda minima* (L.) A.Braun & Asch., *Ipomoea nil* (L.) Roth, *Echinacea purpurea* (L.) Moench, *Periploca forrestii* Schltr., *Arctium lappa* L., *Dipsacus japonicus* Miq., *Valeriana officinalis* L., *Erigeron breviscapus* (Vaniot) Hand.-Mazz., *Eleutherococcus nodiflorus* (Dunn) S.Y.Hu, *Lycium ruthenicum* Murray, *Helianthus tuberosus* L.

Summarizing Table 1, the findings revealed that CGA, NCGA, CCGA, ICGA-A, ICGA-B, and ICGA-C are present in various plants, including *Lonicera japonica* Thunb., *Chrysanthemum morifolium*, *Artemisia scoparia* Waldst. & Kit., *Artemisia argyi* H. Lév. & Vaniot, *Mesua ferrea* L., *Stevia rebaudiana* (Bertoni) Bertoni, *Cuscuta chinensis* Lam., *Akebia quinata*, *Prunus amygdalus* Batsch, *Tussilago farfara* L., *Spatholobus suberectus* Dunn, *Artemisia annua* L., *Eleutherococcus nodiflorus* (Dunn) S.Y.Hu, and *Lycium ruthenicum* Murray. Although these six CGAs are present in these plants, significant differences exist in their specific content ratios. For instance, in Lonicerae Japonicae Flos, the content ratios of CGA (2.77%), CCGA (0.15%), NCGA (0.26%), ICGA-B (0.09%), ICGA-A (2.15%), and ICGA-C (0.35%) vary (Liu et al., 2023b).

The distribution patterns of CGAs reveal that traditional CGA is the most widely distributed in traditional Chinese medicine. Among the novel derivatives, the ICGA class (including three isomers: ICGA-A, ICGA-B, and ICGA-C) exhibits greater diversity in plant sources compared to NCGA and CCGA. Notably, CGAs in plant systems predominantly exist in the form of monocaffeoylquinic acid, with *Phellodendron amurense* Rupr as a typical example. In contrast, some species, such as *Angelica sinensis* (Oliv.) Diels and *Eucommia ulmoides* Oliv., contain both monocaffeoylquinic acid and dicaffeoylquinic acid complex structures. Beyond their distribution, the biosynthesis of CGAs also demonstrates spatiotemporal dynamics, with their content regulated by three primary factors: (1) Geographical environment: A nearly 10-fold difference in the content of 3-CQA is observed between Ethiopia (Wale et al., 2024) and Yunnan coffee-growing regions in China (Luo et al., 2025), which is closely related to soil composition, climate, and cultivation practices; (2) Harvesting season: The content of 4-CQA in Eucommia ulmoides leaves is twice as high in spring as in autumn (Adeosun et al., 2022), likely due to photoperiod and temperature variations; (3) Medicinal parts: The content of CGAs in the woody stems of coffee plants is 75% lower than that in herbaceous stems (Acidri et al., 2020), reflecting the gradient differences in metabolic activity among different organs. These findings provide important insights for optimizing the harvesting strategies of traditional Chinese medicinal materials.

Current research primarily focuses on the correlation between macro-environmental factors, such as geographical location and harvesting season, and the content of CGAs. However, the complete molecular mechanisms by which environmental signals affect metabolic pathways remain unclear, especially the in-depth analysis of the association between soil parameters (e.g., pH) and the expression of core regulatory genes. Two main challenges exist: (1) Traditional univariate analyses fail to reveal the nonlinear regulatory effects of dynamic macro-factors on gene expression; (2) The molecular pathways by which macro-factors regulate CGAs synthase genes through epigenetic modifications or transcription factor networks are still poorly understood. Future research should integrate high-throughput detection and gene-editing technologies to elucidate the precise regulatory networks of key environmental factors, such as soil acidity and humidity, on CGA synthesis genes. Additionally, intelligent models should be developed to quantify the impact of different soil conditions on CGA biosynthesis, providing theoretical support and technical pathways for the targeted enhancement of chlorogenic acid yield.

**FIGURE 2:**
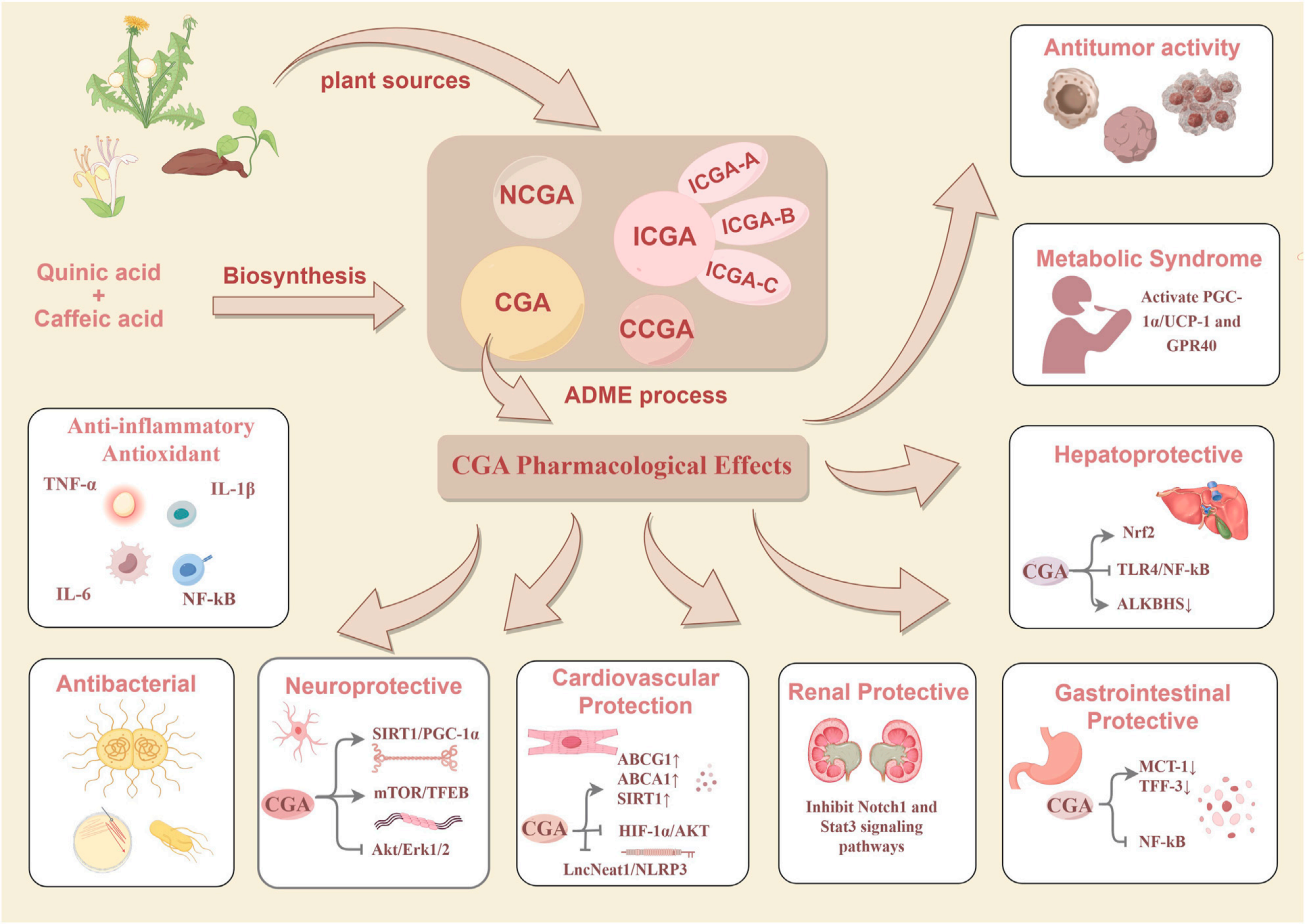
The plant sources of CGAs and the biosynthesis, ADME processes, pharmacological effects of chlorogenic CGA.

In the research field of CGAs, CGA has become the focus due to its extensive pharmacological activities and relatively high content. Current research on CGA has been extensive, covering its biosynthetic pathway (Lu et al., 2024), absorption-distribution-metabolism-excretion (ADME) process (Weerakoon et al., 2021) and pharmacological mechanisms of action (Wang et al., 2022). This study aims to systematically investigate the biosynthetic pathway, ADME characteristics, and pharmacological effects of CGA, providing a theoretical basis and reference for related research. The research framework is illustrated in detail in Figure 2.

## 3 Biosynthetic pathway of CGA

The biosynthesis of CGA is part of the phenylpropanoid metabolic pathway and relies heavily on the specific catalytic actions of the acyltransferase enzyme family. Over the course of evolution, different species have developed distinct synthetic pathways through the functional differentiation of enzyme genes and the evolution of substrate specificity. As of now, a minimum of three biosynthetic routes for CGA have been recognized. The first pathway entails the esterification of caffeoyl-CoA and quinic acid, facilitated by hydroxycinnamoyl-CoA quinate hydroxycinnamoyl transferase (HQT), which is the primary mechanism for CGA production. In 2004, Niggeweg et al. (Niggeweg et al., 2004) successfully cloned the cDNA of HQT from tomato and tobacco and validated through gene silencing experiments that HQT is the principal factor responsible for CGA accumulation in solanaceous plants. Subsequent studies have demonstrated that overexpression of HQT in tomatoes significantly enhances CGA accumulation without significantly altering the levels of other soluble phenolic botanical drugs. Studies have shown that the majority of plants synthesize CGA through pathway 1 (Alcázar Magaña et al., 2021), which has played an important role in the conversion of caffeoyl shikimic acid in angiosperms such as *Arabidopsis thaliana*, *Medicago truncatula*, and *Populus trichocarpa*, and in gymnosperms such as *Larix decidua* (Saleme et al., 2017; Wang et al., 2019). The second pathway is characterized by the cooperative activity of two enzymes: hydroxycinnamoyl-CoA shikimate/quinate hydroxycinnamoyl transferase (HCT) and p-coumaroyl ester 3′-hydroxylase (C3H). Through the interaction between caffeoyl-CoA and quinic acid or shikimate, which is catalyzed by HCT, this route produces intermediate products, which are further transformed into CGA by C3H (Hoffmann et al., 2003). This pathway is the primary route for CGA synthesis in Solanaceae species, including tomato, eggplant, potato, and tobacco. The third pathway involves the conversion of caffeoylglucose to CGA by quinic acid hydroxycinnamoyl transferase. This pathway utilizes caffeoylglucose as a precursor to generate CGA under enzymatic catalysis (Payyavula et al., 2015).

The biosynthesis of CGA is a complex and diverse process. Its metabolic diversity is reflected not only in species-specific enzymatic reaction pathways but also in its close association with multi-tiered transcriptional regulatory networks. Multiple families of transcription factors are crucial in regulating the production of CGAs. These transcription factors can specifically bind to the promoter regions of structural genes implicated in CGA biosynthesis, therefore modulating CGA production by activating or repressing gene expression. The primary transcription factor families involved are the MYB, WRKY, ERF, and bHLH families. In tobacco, the overexpression of the NtMYB59 gene markedly enhances the CGA content in the leaves, suggesting that NtMYB59 positively influences CGA production in tobacco (Zhang et al., 2024). In dandelion, overexpression of TaWRKY14 enhances the CGA content and upregulates the expression of TaPAL1 (Liu et al., 2020a). Similarly, overexpression of NtERF13a in tobacco induces the transcription of the NtHCT gene by directly binding to the GCC box and DRE elements in the NtHCT gene promoter, thereby promoting CGA biosynthesis in tobacco leaves (Wang et al., 2023b). In dandelion, TabHLH1 regulates the expression of structural genes such as TaHQT2, Ta4CL, TaCHI, and TaF3′H and directly binds to the bHLH binding elements in the promoter regions of TaHQT2 and Ta4CL. Studies have demonstrated that the CGA and luteolin contents are significantly elevated in TabHLH1 overexpressing lines (Liu et al., 2021). Table 2 summarizes the key regulatory enzymes, major transcription factors, and their regulatory functions in the three biosynthetic pathways. Additionally, CGA biosynthesis is influenced by various external environmental factors, including plant hormones, light, temperature, water, and inorganic salts. Different species may exhibit differential responses to these external factors, leading to variations in CGA synthesis levels.

**TABLE 2:** Comparative analysis of differences in biosynthetic pathways.

Biosynthetic pathway	Key functional enzymes	Transcription factors	Regulatory role	References
Pathway 1	PAL,C4H,4CL,HCT,C3H,HQT	AtMYB111,CcMYB12b,CcMYB1,LmMYB15,SmMyb1,StAN1,TaWRKY14,TaWRKY14,NtERF13,TabHLH1	CcMYB1: + Others: −	Payyavula et al. (2013), Docimo et al. (2016), Knevitt (2017), Wang et al. (2019), Wang et al. (2023a), Liu et al. (2020a), 2021; Tang et al. (2021)
Pathway 2	PAL,C4H,4CL,HCT,C3H	AtMYB111,CcMYB1,LmMYB15,StAN1,TaWRKY14,TaWRKY14,NtERF13,TabHLH1	CcMYB1: − Others +	Payyavula et al. (2013), Pandey et al. (2014), Knevitt (2017), Liu et al. (2020a), Liu et al. (2021), Tang et al. (2021)
Pathway 3	PAL,UGCT, HCGQT	AtMYB111,StAN1,TaWRKY14,TaWRKY14	+	Payyavula et al. (2013), Pandey et al. (2014), Liu et al. (2020a)

+: Positive regulation, −: Negative regulation.

Abbreviations: PAL, Phenylalanine ammonia-lyase; C4H, Cinnamic acid 4-hydroxylase; 4CL, 4-Coumarate-CoA ligase; HCT, Hydroxycinnamoyl-CoA:shikimate/quinate hydroxycinnamoyltransferase; C3H, p-Coumarate 3-hydroxylase; HQT, Quinate hydroxycinnamoyltransferase; UGCT, UDP-glucose:cinnamate glucosyltransferase; HCGQT, Hydroxycinnamoyl D-glucose:quinate hydroxycinnamoyltransferase.

## 4 The ADME process of CGA

There is a significant relationship between the biosynthetic pathway of CGA in plants and its metabolic process in the human body. The structural and chemical features of CGA are determined by the biosynthesis pathway in plants, however, the bioavailability and biological activity of CGA are directly influenced by the metabolism process in the human body.

### 4.1 Absorption of CGA

#### 4.1.1 *In vitro* absorption model study

Olivier Mortelé's team demonstrated through the Caco-2 cell model that both CGA and quinic acid exhibit active efflux during intestinal absorption, suggesting a relatively low overall absorption rate for CGA (Mortelé et al., 2021). Mechanistic studies have shown that dietary fats (such as soybean oil and coconut oil) can significantly enhance the permeability of CGA in the Caco-2 monolayer by increasing cell membrane fluidity (Weerakoon et al., 2021), providing *in vitro* evidence supporting the enhanced bioavailability of CGA in oil-containing diets.

#### 4.1.2 *In vivo* absorption characteristics

Clinical pharmacokinetic data indicate that oral CGA exhibits rapid absorption characteristics in the human body, with a T_max_ of approximately 1 h and a t_1/2_ ranging from 1 to 2 h (Yang et al., 2020). Moreover, different routes of administration, including oral, intramuscular injection, and intravenous infusion, do not affect T_max_ and t_1/2_. The C_max_ and AUC_0→inf_ of CGA are positively correlated with the administered dose. Following oral administration of CGA, the acidic environment in the stomach helps maintain the structural stability of CGA, with approximately one-third of the dose entering the blood system through passive diffusion in the small intestine, while the remaining two-thirds of CGA continues to move forward, eventually reaching the cecum and colon (Olthof et al., 2001). The bioavailability of CGA following intramuscular injection is significantly higher than that following oral administration. Notably, significant differences in pharmacokinetic parameters between humans and animals (such as rats or rabbits) may lead to interspecies differences in bioavailability.

### 4.2 Distribution of CGA

Upon entering the bloodstream, CGA interacts with the specific IIA binding site of human serum albumin (HSA), and the molar ratio of CGA to HSA determines how strong this interaction is. One can notice a 1:1 binding ratio when the molar ratio of CGA to HSA falls within the range of 1:10. The binding ratio, however, rises to 2:1 when the molar ratio reaches 10:30 (Kang et al., 2004). In animal models, the distribution of CGA is widespread, with the highest concentration detected in the kidneys. Relatively higher levels of CGA are found in the lungs, heart, muscles, spleen, and stomach, while lower levels are observed in the testes, brain, and intestines (Chen et al., 2017).

### 4.3 Metabolism and excretion of CGA

Methylation, reduction, and phase II processes, including glucuronidation and sulfation, are among the metabolic changes that CGA goes through in the liver (Moridani et al., 2001). These processes are essential for the detoxification and excretion of CGA and its metabolites. A study on repeated intravenous injection of CGA in rats revealed that it is primarily converted into NCGA, CCGA, methylated derivatives of CGA, and methylated derivatives of CCGA within the body. These metabolites are predominantly excreted via urine (Gonthier et al., 2003). However, the oral bioavailability of CGA is low, mainly due to its hydrophilicity and extensive metabolism into various metabolites, including caffeic acid and quinic acid (Trivedi and Puranik, 2023). As a result, the systemic biological effects of CGA are highly dependent on its metabolism and transformation by the gut microbiota in the colon. This biotransformation process begins with the hydrolysis of the ester bond of CGA by bacterial esterases, yielding caffeic acid and quinic acid (Plumb et al., 1999). Following this initial hydrolysis, a series of complex microbial reactions occur: caffeic acid is further metabolized into highly bioactive secondary metabolites, such as dihydrocaffeic acid, ferulic acid, isoferulic acid, coumaric acid, and various phenylpropanoid derivatives. Meanwhile, quinic acid can be degraded into benzoic acid and subsequently transformed into products like hippuric acid (Clifford et al., 2017).

The absorption and metabolic fate of these microbial metabolites are significantly different from those of the parent compound. Notably, dihydrocaffeic acid and other hydrogenated derivatives exhibit superior membrane permeability compared to CGA itself, allowing them to be more effectively absorbed into the portal vein circulation (Clifford et al., 2017). These absorbed metabolites then undergo hepatic conjugation reactions and are ultimately excreted in urine. This key dependence on microbial messtabolic pathways implies that the differences in the composition of gut microbiota among individuals are the primary determinants of the final efficacy and health benefits of CGA, forming the basis of its “gut-axis” effect.

### 4.4 Strategies for enhancing the bioavailability of CGA

CGA exhibits a broad spectrum of biological activities; however, its clinical application is hindered by several pharmacokinetic limitations, including low bioavailability, poor chemical stability, and a short half-life *in vivo*. To address these challenges, various novel drug delivery systems based on pharmaceutical science have been developed in recent years. These systems aim to enhance the biological efficacy of CGA by improving its solubility, stability, and targeting capabilities.

#### 4.4.1 Nanodelivery systems

Nanodelivery systems have emerged as a current research focus. These systems encapsulate CGA using nanocarriers, which effectively protect its stability in the gastrointestinal environment (Sánchez-Moreno et al., 2018). The nanoscale effect is utilized to enhance mucosal adhesion and cell penetration, thereby enabling controllable or targeted drug release (Raman et al., 2015). For example, Li et al. (2022) used poly (lactic-co-glycolic acid) (PLGA) to prepare nanoparticles that exhibit good tissue penetration and retention properties, achieving sustained release under physiological pH conditions. Yao et al. (2023) employed tetrahedral framework nucleic acid (tFNA) as a carrier, which significantly improved the stability of CGA in serum and extended the release period to over 24 h, thereby enhancing its anti-fibrotic activity.

#### 4.4.2 Phospholipid complex technology

Lipid-based systems primarily enhance the lipophilicity of CGA to promote its transmembrane transport. Feng et al. (2016) prepared CGA liposomes (CAL), which increased its relative bioavailability by 1.29 times. Further functional modifications can enhance targeting. Ye et al. (2020) developed mannosylated liposomes that specifically target M2-type tumor-associated macrophages, thereby enhancing local efficacy and inhibiting the growth of G422 glioblastoma.

#### 4.4.3 Biomacromolecule carrier systems

Biomacromolecule carriers have demonstrated unique advantages in protecting CGA and achieving local controlled release. Hydrogel systems can maintain the stability of CGA during processing and storage and achieve environment-responsive or colon-specific release. This enhances the interaction between CGA and gut microbiota, thereby improving the health benefits of its microbial metabolites. Guo et al. (2022) used sodium alginate composite hydrogel dressings to achieve sustained release of CGA, resulting in a wound healing rate exceeding 98% on day 11. Other studies have constructed supramolecular hydrogels using the self-assembly behavior of CGA, which exhibit good biocompatibility and self-healing ability, providing new ideas for local treatment (Huang et al., 2023). Additionally, emulsion systems have demonstrated high encapsulation efficiency and good stability (Dima and Dima, 2018).

In summary, a variety of strategies, including nanotechnology, lipid carriers, and biomacromolecule delivery systems, can be employed to improve the stability, solubility, absorption pathways, and targeting of CGA. These improvements provide significant support for its further application in the fields of medicine, functional foods, and biomaterials by enhancing its therapeutic potential and overall efficacy.

## 5 Pharmacological effects of CGA

### 5.1 Anti-inflammatory and antioxidant effects

Inflammation is a reaction caused by infection, tissue damage, or harmful stimuli. It is a complex physiological and pathological process characterized by redness, swelling, heat, pain, and functional disorders (Medzhitov, 2021). If left unchecked, it may progress to various chronic inflammatory diseases. These diseases are characterized by a prolonged course and a high propensity for relapse. Currently, antibiotics are commonly used for anti-inflammatory treatment. However, their use can lead to the development of drug resistance in patients, cause damage to vital organs such as the liver, kidneys, and induce adverse reactions such as allergies (Nanayakkara et al., 2021).

CGA possesses natural antioxidant activity and is widely employed in experimental research on inflammatory diseases. Its activity is determined by its molecular structure. The CGA molecule contains five active hydroxyl groups and one carboxyl group. These groups are derived from the esterification reaction between caffeic acid and quinic acid, resulting in a unique structure that features three active groups: ester bonds, unsaturated double bonds, and dihydroxyphenols (Miao and Xiang, 2020). The phenolic hydroxyl groups can combine with free radicals to form antioxidant-active hydrogen radicals, effectively neutralizing hydroxyl radicals and superoxide anions, thus displaying significant antioxidant properties. According to studies, CGA’s polyhydroxy structure can shield thiol groups from hydroxyl groups’ attacks, encouraging the creation of intramolecular or intermolecular disulfide bonds instead. Furthermore, proteins and CGA can create protein/polyphenol complexes by hydrogen bonding, which boosts proteins’ antioxidant potential and stops free radicals from spreading (Tarahi et al., 2024). However, the enone double bond in the CGA molecule exhibits strong electrophilicity, which may lead to covalent binding with protein thiol groups and potentially cause allergic adverse reactions (Bariana et al., 1965). It is important to note that CGA often contains a catechol moiety, which may lead to its classification as a pan-assay interference compound (PAINS). Compounds with active groups such as catechol, quinone, or Michael acceptors are prone to causing false-positive results. Traditional methods are limited in their ability to capture the subtype-specific interface of CGA. To address this limitation, the GRIP method screens for subtype-specific homologous sequences (Nemoto and Saito, 2021). CGA’s anti-inflammatory properties are closely related to its antioxidant activity. Tumor necrosis factor (TNF-α), prostaglandin E2 (PGE2), nitric oxide (NO), interleukin-1β (IL-1β), interleukin-6 (IL-6), and interleukin-8 (IL-8) are among the inflammatory mediators whose synthesis and secretion are modulated by CGA. Furthermore, it protects cells and tissues from harm by controlling important signaling pathways and associated factors such as nuclear factor κB (NF-κB), mitogen-activated protein kinase (MAPK), and nuclear factor erythroid 2-related factor 2 (Nrf2). As shown in Table 3, CGA has great therapeutic promise for the treatment of a number of illnesses because of its ability to efficiently control inflammatory mediators and reduce oxidative stress. These results offer a strong theoretical basis for the future advancement and use of CGA in pharmaceutical and clinical contexts.

**TABLE 3:** Anti-inflammatory and antioxidant mechanisms of chlorogenic acid in therapeutic applications.

Disease types	Disease name	Types	Model	Dose and treatment duration	Mechanism of action	References
Nervous System Diseases	Parkinson’s disease (PD)	*in vivo* *in vitro*	animal: A53T (homozygous) C57BL mice cell: 6-OHDA-induced primary murine neurons and SH-SY5Y cells model	vivo: 50 mg/kg for 21 days vitro: 50 μM for 24 h	Removal of excess ROS in PD models, inhibition of Erk1/2 activation, and prevention of Akt inactivation	He et al. (2024)
	Alzheimer’s disease (AD)	*in vivo*	APP/PS1 double transgenic C57BL/6 mice	80 mg/kg for 8 weeks	Activation of the SIRT1/PGC-1α/BACE1 signaling pathway	Shi et al. (2023a)
Cardiovascular System Diseases	Atherosclerosis	*in vitro*	oxLDL-induced HUVECs cells model	25, 50, 100 μM for 24 h	SIRT1 activity↑, AMPK–α activation↓, PGC-1α expression↓	Tsai et al. (2018)
	Heart failure	*in vivo* *in vitro*	animal: TAC-induced C57BL/6 mice cell: H9c2 cardiomyocyte cells model	vivo: 58 mg/kg,116 mg/kg for 6 weeks vitro: 1–100 μmol/L for 48 h	Expression of ferroptosis-related proteins↑,SLC7A11↑, GPX4↑cardiac function↑,lipid peroxidation↓, ROS levels↑,activation of SLC7A11/GPX4 signaling pathway	Huang et al. (2024)
	Ischemic heart disease	*in vivo* *in vitro*	animal: MIRI C57BL/6 mice cell: Hypoxia/reoxygenation HL-1 cells model	vivo: 30 mg/kg CGA for 4 weeks vitro: 0.2, 2 μM CGA for 12 h	LncNeat1 expression levels↓,inhibition of NLRP3 inflammasome activationto prevent myocardial ischemia-reperfusion injury (MIRI)	Chai et al. (2023)
Urinary System Diseases	Renal fibrosis	*in vivo* *in vitro*	animal: UUO-induced C57BL/6 mice cell: TGF-β1-induced HK-2 cells model	vivo: 40 mg/kg, 80 mg/kg for 10 days vitro: 50, 100 Mm for 24 h	Regulation of the TLR4/NF-қB signaling pathway	Jiao et al. (2024)
	Acute kidney injury (AKI)	*in vivo*	Cisplatin-induced Wistar rats	20 mg/kg for 2 weeks	Inhibition of the TLR4/NLRP3 and caspase-1/GSDMD signaling pathways	Badr et al. (2023)
	Diabetic nephropathy	*in vivo* *in vitro*	animal: HFD/STZ-induced Wistar rats cell: HG-stimulated HK-2 cells model	vivo: 10 mg/kg for 10 weeks vitro: 20, 50, 100 μM for 48 h	Activation of the Nrf2 pathway and inhibition of NLRP3 inflammasome activation	Bao et al. (2025)
Digestive System Diseases	Colitis	*in vivo*	DSS-induced ICR mice	250, 500 mg/kg CGA for 3 weeks	Activation of the Nrf-2/HO-1 pathway to inhibit oxidative stress and inflammatory responses, and promote intestinal barrier function	Wan et al. (2021)
	Alcoholic steatohepatitis (ASH)	*in vivo*	HFD/Alcohol-induced Wistar rats	40 and 80 mg/kg, 8 weeks	Regulation of genes encoding CYP2E1/Nrf2 and TLR4/NF-κB to improve ethanol-induced oxidative stress and inflammation	Buko et al. (2021)
	Hepatic fibrosis	*in vivo* *in vitro*	animal: CCl_4_-induced SD rats cell: PDGF-stimulated HSC-T6 cells model	vivo: 60 mg/kg for 8 weeks vitro: 12.5, 25, 50 μg/mL for 2 h	1 CYP2E1 expression↓, Nrf2↑HO-1↑, GCLC↑, NQO1↑, MDA levels↓GSH↑, SOD↑, CAT↑ 2 Inhibition of the TLR4/MyD88/NF-κB signaling pathway	Shi et al. (2013), 2016
	Cholestatic liver disease (CLD)	*in vivo*	ANIT-induced 129/sv mice	50 mg/kg for 5 days	STAT3 and NFκB signaling pathways↓,inhibit α-naphthyl isothiocyanate (ANIT)-induced intrahepatic cholestasis and liver injury	Tan et al. (2016)
Endocrine System Diseases	Diabetes	*in vivo*	animal: HG-induced C57BL/KsJ and db/db C57BL/KsJ mice	normal chow containing 0.02% CGA for 12 weeks	Activation of the Nrf2 antioxidant pathway to improve endothelial dysfunction in diabetic mice	Wang et al. (2021a)

Abbreviations: ROS, Reactive oxygen species; ERK1/2, Extracellular signal-regulated kinases 1 and 2; Akt, Serine/threonine kinase; SOD, Superoxide dismutase; GSH, Glutathione; HO-1, Heme oxygenase 1; MDA, Malondialdehyde; Keap1, Kelch-like ECH-associated protein 1; iNOS, Inducible nitric oxide synthase; COX-2, Cyclooxygenase-2; PINK1, PTEN-induced kinase 1; Parkin, Ubiquitin ligase; IL-18, Interleukin-18; IFN-γ, Interferon-γ; EGFR, Epidermal growth factor receptor;SLC7A11, Solute carrier family 7 member 11; GPX4, Glutathione peroxidase 4; NLRP3, NLR family pyrin domain-containing 3; TLR4, Toll-like receptor 4; GSDMD, Gasdermin D; NQO1, NAD(P)H, quinone oxidoreductase 1; PI3K, Phosphoinositide 3-kinase; MPO, Myeloperoxidase; DSS, Dextran sodium sulfate; Caveolin-1, Caveolin-1; STAT3, Signal transducer and activator of transcription 3; GCLC, Glutamate-cysteine ligase catalytic subunit; CAT, Catalase; MyD88, Myeloid differentiation factor 88; ANIT, α-Naphthyl isothiocyanate; CYP2E1, cytochrome P450 2E1 enzyme.

### 5.2 Antibacterial effects

In clinical practice, the proliferation of multidrug-resistant bacteria has emerged as a leading cause of mortality among infected patients. Consequently, the development of new antibiotics has become a prominent topic of research. More and more scientists are now focusing on CGA’s antibacterial properties against clinical bacteria and strains that are resistant to multiple drugs. These mechanisms can be categorized into three primary aspects: (1) Disruption of physical barriers, achieved by increasing cell membrane permeability, interfering with biofilm formation (Yu et al., 2024), and compromising structural integrity (Chen et al., 2022a); (2) Regulation of metabolic pathways via the inhibition of pivotal enzymes in energy metabolism and the disruption of quorum sensing systems (Zhang et al., 2023a); (3) Induction of oxidative stress: facilitating the buildup of reactive oxygen species and expediting bacterial death. The antibacterial efficacy of CGA is contingent upon dosage and duration, while the antibacterial properties of its metabolites, including caffeic acid and gallic acid, are markedly superior to those of the parent molecule (Luís et al., 2014). This finding offers new insights for structural modification.

In addition to its antibacterial effects, CGA also demonstrates multi-target characteristics in fungal inhibition. *In vitro*, experiments have confirmed that CGA can inhibit the germination of spores and the elongation of germ tubes of *Botrytis cinerea*. The underlying mechanism may involve reducing ergosterol synthesis, inducing the accumulation of malondialdehyde (MDA), and triggering lipid peroxidation in the fungal membrane, ultimately leading to hyphal disintegration (Dai et al., 2024a). This stage-specific mode of action endows CGA with unique application potential in post-harvest preservation of fruits and vegetables.

### 5.3 Neuroprotective effects

Neurodegenerative diseases are conditions characterized by the progressive deterioration of neuronal function and myelin sheaths in the brain, typically associated with aging and manifesting as cognitive impairment, involuntary movements, gait abnormalities, and bradykinesia (Gonzales et al., 2022). Neurodegenerative illnesses, like Alzheimer’s disease (AD) and Parkinson’s disease (PD), are well-established as prevalent conditions impacting the nervous system (Weintraub et al., 2022). At present, effective therapies for these disorders remain constrained, considerably impacting patients’ quality of life and health. Recent research indicates that a combination of moderate aerobic exercise and CGA treatment can enhance cognitive function in mice. The impact is attained by the activation of the SIRT1/PGC-1α signaling pathway, which subsequently mitigates oxidative stress, neuroinflammation, and β-amyloid (Aβ) accumulation (Shi et al., 2023a). The combined intervention of Comprehensive CGA and exercise is more efficacious than employing either method in isolation. A further study examined the impact of CGA on Aβ^25-35^-induced injury in human neuroblastoma cells (SH-SY5Y) and cognitive impairments in the APP/PS1 mice model. The results showed that CGA enhanced lysosomal function by activating the mTOR/TFEB signaling pathway, thereby inhibiting the autophagy process and improving cognitive deficits (Gao et al., 2020). In summary, CGA may protect against AD through multiple mechanisms, including regulating the autophagy process, enhancing lysosomal function, improving cognitive function, and exerting anti-inflammatory and antioxidant activities. CGA can inhibit neuronal death in Parkinson’s disease by regulating the Akt/Erk1/2 signaling pathway. In Parkinson’s disease experimental models, CGA pretreatment markedly diminished ROS caused by PD toxins, blocked Erk1/2 activation, prevented Akt inhibition, and averted neuronal cell death (He et al., 2024). Furthermore, CGA mitigated gait impairments in Parkinson’s disease model mice, with its neuroprotective effects validated in various endocrine areas and the substantia nigra. Subsequent research has demonstrated that CGA can mitigate PD symptoms by enhancing autophagy. In zebrafish models, CGA mitigated Parkinson’s disease-like symptoms generated by 1-methyl-4-phenyl-1,2,3,6-tetrahydropyridine (MPTP), encompassing the degeneration of dopaminergic neurons and blood vessels, diminished locomotion, and apoptotic occurrences in the brain. CGA also modulated the expression of genes related to Parkinson’s disease and autophagy, including *α-synuclein (α-syn)*, LC3B, SQSTM1, ATG5, ATG7, and ULK1B, thereby indicating its capacity to enhance autophagy in Parkinson’s disease pathogenesis (Gao et al., 2023). Therefore, CGA may become a candidate drug for anti-PD treatment, with its mechanism of action possibly related to promoting autophagy and modulating the Akt/Erk1/2 signaling pathway (Zhang et al., 2022). It should be emphasized that most of these studies are based on preclinical research, and their efficacy and safety in patients with Parkinson’s disease require further validation through clinical trials. In addition to its potential therapeutic effects on AD and PD, CGA has also been found to improve PTSD-like symptoms such as anxiety and depression (Chen et al., 2021). These results set the scientific groundwork for CGA’s future development as a therapeutic medication and offer a significant theoretical basis for its use in neuropsychiatric disorders and neurodegenerative diseases.

### 5.4 Cardiovascular protection

Cardiovascular diseases encompass a range of conditions, including atherosclerosis, hypertension, myocardial infarction, and heart failure (Fang et al., 2023). In the context of atherosclerosis prevention and treatment, CGA has been shown to inhibit disease progression by promoting cholesterol efflux (Soltani et al., 2021). In particular, CGA promotes the production of ABCG1 and ABCA1, two molecules linked to cholesterol transport, which helps RAW264.7 macrophages release lipids (Wu et al., 2014). One important process in the development of atherosclerosis and a critical predictor of cardiovascular disorders is endothelial dysfunction. According to a recent study, CGA increases SIRT1 to reduce oxidative stress brought on by oxidized low-density lipoprotein (ox-LDL). Additionally, by modifying the AMPK/PGC-1 signaling pathway, CGA reduces mitochondrial dysfunction and endothelial oxidative stress brought on by oxLDL (Tsai et al., 2018). In the context of hypertension management, research has demonstrated that CGA effectively suppresses catecholamine (CA) release from adrenal medullary chromaffin cells through dual mechanisms. First, CGA decreases the amount of Na^+^ and Ca^2+^ that enter the cell through ion channels. Second, it inhibits the release of Ca^2+^ from the cytoplasmic calcium storage by activating nitric oxide synthase (NOS), which increases the release of nitric oxide (NO). Cholinergic and AT1 receptor activation results in CA secretion, which is suppressed by these effects. Blocking AT1 and cholinergic receptors in neurons seems to be linked to CGA’s inhibitory actions. These results suggest that CGA consumption may help prevent or treat cardiovascular disorders by lowering the secretion of CA by the adrenal glands, which in turn lowers the levels of CA in the blood and aids in the control of hypertension (Lim and Koh, 2023). Furthermore, CGA has been shown to inhibit ACE activity and smooth muscle cell proliferation, block the HIF-1α/AKT signaling pathway, and induce vascular remodeling (Lukitasari et al., 2020). The metabolites of CGA can reduce oxidative stress and achieve antihypertensive effects by improving vascular endothelial function and increasing NO bioavailability (Zhao et al., 2012). By preventing pyroptosis, which is mediated by the long non-coding RNA Neat1 (LncNeat1)/NLRP3 inflammasome, CGA also demonstrates strong anti-inflammatory properties that protect against myocardial ischemia-reperfusion injury (MIRI). This lowers the inflammatory cascade and offers protection against MIRI (Chai et al., 2023). Another study demonstrated that CGA treatment alleviated isoproterenol-induced cardiac hypertrophy in rats by regulating S1pr1 and thereby activating the AMPK/SIRT1 pathway (Ping et al., 2024). Additionally, CGA increases the secretion of angiopoietin-2, enhances endothelial cell barrier function, and promotes angiogenesis, all of which have beneficial effects on the cardiovascular system (Wuttimongkolchai et al., 2022). Takuya Watanabe et al. (Watanabe et al., 2006)demonstrated in a placebo-controlled randomized clinical trial that CGA effectively lowers blood pressure and is safe for patients with mild hypertension. In conclusion, CGA has the potential to serve as a therapeutic agent for cardiovascular diseases because it exhibits a variety of cardiovascular protective properties through mechanisms including cholesterol efflux, oxidative stress reduction, and regulation of important signaling pathways.

### 5.5 Renal protective effects

The combined effects of these several mechanisms underlie CGA’s function in renal protection. CGA dramatically reduced kidney damage, inflammation, oxidative stress, and fibrosis in a rat model of unilateral ureteral obstruction (UUO), according to a study by Jiao et al. (2024). CGA successfully suppressed the oxidative stress process and the production of inflammatory factors in both *in vitro* and *in vivo* fibrosis models. Network pharmacology, transcriptomic analysis, and molecular docking further showed that the TLR4/NF-κB signaling pathway is regulated to mediate CGA’s anti-fibrotic actions. Furthermore, by blocking the Notch1 and STAT3 signaling pathways, CGA lessens renal lipid accumulation and improves renal fibrosis in diabetic nephropathy (Yang et al., 2024). Collectively, these results show that CGA protects the kidneys through a variety of routes, with a focus on fibrotic and inflammatory signaling pathway modulation. Clarifying the clinical significance of these pathways and determining the ideal therapeutic dosage should be the main goals of future research.

### 5.6 Gastrointestinal protective effects

CGA uses a variety of methods to provide notable gastrointestinal protection. The release of pro-inflammatory cytokines, such as TNF-α and IL-6, is significantly decreased by CGA through downregulating CD14 expression and blocking the NF-κB signaling pathway. This activity maintains the shape and function of the cell membrane, improves intestinal barrier function, and helps preserve the integrity of tight junctions in intestinal epithelial cells (Yu et al., 2021a). Additionally, CGA works with the gut microbiota and its metabolites to alleviate post-infectious irritable bowel syndrome (PI-IBS) (Zheng et al., 2023). Additionally, CGA increases intestinal damage repair, decreases MCT-1 and TFF-3 expression, and suppresses NF-κB expression, suggesting that it has positive effects on ulcerative colitis caused by dextran sulfate sodium (Maslin et al., 2022). Ultimately, CGA safeguards the intestinal barrier via three principal mechanisms: (1) inhibiting NF-κB inflammatory signaling, (2) regulating microbial metabolic activity, and (3) promoting mucosal repair. However, the existing studies are predominantly based on rodent models and do not provide sufficient data regarding the human intestinal immune microenvironment.

### 5.7 Hepatoprotective effects

Studies have demonstrated that CGA plays a significant role in improving various types of liver diseases, including drug-induced liver injury (DILI), ALD, metabolic-associated fatty liver disease (MAFLD), CLD, liver fibrosis, and liver cancer. CGA exhibits significant antioxidant and anti-inflammatory effects by activating Nrf2 and inhibiting the Toll-like receptor 4 (TLR4)/NF-κB signaling pathway. It has also been discovered that the benefits of CGA for a number of liver disorders involve important molecules like AMPK and ERK1/2 as well as vital physiological functions including the gut barrier and gut microbiota (Xue H. et al., 2023). By blocking the activity of the RNA demethylase AlkB homolog 5 (ALKBH5), studies have also demonstrated that CGA can control autophagy and reduce hepatic steatosis (Meng et al., 2023). Furthermore, CGA prevents liver fibrosis caused by schistosomiasis via altering the interleukin-13 (IL-13)/miR-21/Smad7 signaling relationship in the hepatic stellate LX2 cell line (Wang et al., 2017). Therefore, CGA shows promise as an anti-fibrotic drug to treat liver fibrosis caused by schistosomiasis. Through the Nrf2 antioxidant route, the TLR4/NF-κB anti-inflammatory pathway, and the ALKBH5-autophagy regulatory network, CGA generally has multi-target hepatoprotective effects. However, the cell-specific effects of ALKBH5 inhibition and the metabolic adaptations associated with long-term administration remain to be elucidated.

### 5.8 Metabolic syndrome

The symptoms of metabolic syndrome (MetS) include high triglyceride levels, hyperglycemia, and abdominal obesity. CGA has demonstrated multiple effects in improving metabolic syndrome, including anti-obesity effects, lipid regulation, and glucose metabolism improvement. CGA mitigates hepatic steatosis and reduces blood lipid levels by modulating the PGC-1α/UCP-1 signaling pathway, thereby preventing metabolic disorders associated with glucose metabolism (Zhong et al., 2020). CGA can prevent endotoxemia and glucose metabolism problems, lower the relative weight of visceral and subcutaneous fat, increase the integrity of the intestinal barrier, and decrease weight gain brought on by a high-fat diet (HFD) (Ye et al., 2021). CGA has a 29% affinity for G protein-coupled receptors (GPCRs), and by activating signal transduction pathways, it may be able to stimulate GPR40 to treat diabetes. When GPR40 is activated, the IP^3^ receptor pathway is triggered, which increases intracellular calcium ([Ca^2+^]_i_) and insulin release (Sanchez et al., 2017). CGA inhibits ceramide accumulation to suppress the hepatic insulin response (Xiao et al., 2023). Additionally, in improving anti-diabetic effects, the microbiota may be a key mediator of these beneficial effects (Yan et al., 2022). To encapsulate, CGA ameliorates MetS through three primary mechanisms: promoting adipose browning, enhancing insulin secretion, and modulating the microbiota-metabolite axis.

### 5.9 Antitumor activity

CGA has been evaluated in Phase I clinical trials, and the studies have shown that it effectively treats recurrent high-grade glioma, exhibiting good safety and tolerability (Kang et al., 2023). In addition to glioma, CGA also demonstrates significant preventive and therapeutic effects on various types of tumors, such as lung cancer (Mao et al., 2023), liver cancer (Jiang et al., 2021; Zhou et al., 2021), breast cancer (Zeng et al., 2021), and colorectal cancer (Ranjbary et al., 2023; Pethanasamy et al., 2024). CGA’s anticancer mechanisms include immunological modulation, reversal of multidrug resistance, suppression of telomerase activity, induction of apoptosis, prevention of angiogenesis and metastasis, cell cycle arrest, and inhibition of proliferation. The mechanisms of action of CGA in various cancers are summarized in detail in Table 4, which also offers crucial references for additional study and use.

**TABLE 4:** Mechanisms of antitumor effects of CGA.

Disease	Types	Model	Dose and treatment duration	Mechanism of action	References
Lung Cancer	*in vivo* *in vitro*	animal: BALB/c-nu mice cell: A549 cell	animal: 120 mg/kg for 4 weeks cell: 400, 800 μM for 24 h	Binds to annexin A2, reduces the expression of anti-apoptotic genes downstream of NF-κB, and inhibits the proliferation of A549 cells.	Wang et al. (2020), Mao et al. (2023)
Liver Cancer	*in vivo* *in vitro*	animal: BALB/c-A-nu mice cell: HuH-7,HepG2,MHCC97H, MHCC97L	animal: 120, 480 mg/kg for 35 days cell: 250, 500, 1,000 μM for 48 h	① DNMT1 protein expression↓,inhibiting the proliferation, colony formation, invasion, and metastasis of HepG2 cells, activity of p53 and p21↓, MMP-2↓ MMP-9↓	Liu et al. (2020c), Jiang et al. (2021)
	*in vitro*	PLC/PRF/5, HepG2 cell	cell: 100, 200, 400 μM for 48 h	② Enhances apoptosis by activating pro-apoptotic proteins (annexin V, Bax, and caspase 3/7) and inhibiting anti-apoptotic proteins (Bcl2 and Bcl-xL). inhibit the MAPK and PI3K/Akt/mTORC signaling pathways.	Refolo et al. (2018), Zhou et al. (2021)
Breast Cancer	*in vitro*	MDA-MB-231, MCF-7 cell	25, 50, 100 μg/mL for 6, 12, 24, 48 h	① Bax/Bcl-2↑,p53↑,caspase-3↑,LRP6↓	Changizi et al. (2021), Xue et al. (2023b)
	*in vivo* *in vitro*	animal: Balb/c mice cell: 4T1, EMT6, BT-549, MDA-MB-231 cell	animal: (RLT-03)20.0 mg/g cell: 1.0, 1.5, 2.0, 2.5, 3.0 mg/mL for 24, 48 h	② RTK ligands↓,VEGF↓, EGF↓, CD34↓, IL-10↓, TGF-β↓,	Li et al. (2020)
Colon Cancer	*in vivo* *in vitro*	animal: BALB/c mice cell: HCT116, CT26, HCT15 cell	animal: (LASNB)2.61 g/kg for 18 days cell: 1.25, 2.5, 3.0, 4.0 mg/mL for 24, 48, 72 h	①Inhibits the activation of the receptor tyrosine kinase- (RTK-) MEK-ERK and NF-κB pathways.	Li et al. (2021c), Pethanasamy et al. (2024)
	*in vitro*	SW480, HT-29 cell	250, 500, 1,000, 2000 μM for 24 h	② Bcl-2↓,NF-κB↓,caspase 3↑,caspase 9↑,ROS↑	Ranjbary et al. (2023), Vélez-Vargas et al. (2023)
Pancreatic Cancer	*in vivo* *in vitro*	animal: nude mice cell: PANC-1, CFPAC-1 cell	animal: 80 mg/kg for 12 days cell: 50, 100, 200, 400, 800 μM for 48 h	TFR1↓, inhibits aerobic glycolysis, inhibits the viability of pancreatic cancer cells (PANC-1 and CFPAC-1) and blocks cell cycle progression by arresting the G1 phase.	Yang et al. (2021), Chen et al. (2024)
Leukemia	*in vitro*	U937 cell	50, 100, 150, 200 μM for 48 h	① ΔΨm↓, activation of the caspase-3 pathway↑,apoptosis↑	Yang et al. (2012)
	*in vivo* *in vitro*	animal: nude mice cell: K562, KU812, KCL22, THP-1, U937, REH, Molt 4 cells	animal: 25, 100,150 mg/kg for 10 days vitro: 10 μg/mL for 24 h	② ROS↑, Bcr-Abl phosphorylation↓, apoptosis↑	Bandyopadhyay et al. (2004)
Esophageal Squamous Cell Carcinoma (ESCC)	*in vivo* *in* *vitro*	animal: NOD/SCID mice cell: KYSE30/70/140/150/180/510 cell	animal: 50 mg/kg for 6 weeks cell: 200 μM for 48 h	proliferation of ESCC cells↓, BMI1↓,SOX2↓, MMP-2↓, MMP-9↓	Zhan et al. (2020), Chen et al. (2022b)
Melanoma	*in vivo* *in vitro*	animal: C57BL/6 mice cell: RAW264.7, B16F10 cell	animal: 40 mg/kg for 12 days cell: 50 μg/mL for 24 h	CD8+T/M1-TAM↑growth of B16F10 melanoma cell↓	Li et al. (2021b)

Abbreviations: MMP-2, matrix metalloproteinase-2; DNMT1, DNA methyltransferase 1; mTORC, mammalian target of rapamycin complex; RTK, receptor tyrosine kinase; VEGF, vascular endothelial growth factor; EGF, epidermal growth factor; TGF-β, transforming growth factor-β; IL-10, interleukin-10; CD4^+^, CD4-positive T cells; CD8^+^, CD8-positive T cells; MEK, mitogen-activated protein kinase kinase; ΔΨm, mitochondrial membrane potential; ERK, extracellular signal-regulated kinase.

### 5.10 Other pharmacological effects

In addition to the aforementioned pharmacological effects, recent studies have demonstrated that CGA exhibits multiple other biological activities (Nguyen et al., 2024). CGA may be a good treatment for osteoporosis because it has been shown to increase osteoblast development and proliferation while inhibiting osteoclast formation (Ho et al., 2024). Through a p38 mitogen-activated protein kinase-dependent mechanism, CGA inhibits Bcr-Abl tyrosine kinase and causes chronic myeloid leukemia cells to undergo apoptosis (Bandyopadhyay et al., 2004). In PCOS rats, CGA improves follicular development, hormone levels, and oxidative stress in addition to reducing the clinical symptoms of PCOS via modifying the hypoxia-inducible factor 1α (HIF-1α) signaling pathway, suggesting that it may be used as a treatment for PCOS (Zhang et al., 2023b).

CGA has been proven to possess a variety of pharmacological activities, including antioxidant, anti-inflammatory, and neuroprotective effects, with its mechanisms of action illustrated in Figure 2. It is worth noting that with the widespread application of CGA in pharmaceuticals and functional foods, its safety evaluation has gradually become a research focus. Existing evidence indicates that CGA itself is not a direct allergen; however, there have been cases of hepatic and renal damage associated with the clinical use of some traditional Chinese medicine injections containing CGA(Li et al., 2010). Zhuang Kang et al. observed in a Phase I clinical trial in glioma patients that CGA injection did not induce severe toxic reactions, suggesting good tolerability with short-term use (Kang et al., 2023). Takuya Watanabe’s team further confirmed through a randomized controlled trial that CGA significantly reduced blood pressure in patients with mild hypertension without producing significant safety signals (Watanabe et al., 2006). Despite these findings, the long-term toxicity of CGA and its interactions with complex formulations remain unclear. Based on current evidence, there is an urgent need to establish a comprehensive safety evaluation system that includes pharmacokinetics and toxicological pathology to provide a scientific basis for the precise clinical application of CGA.

## 6 Other CGAs’ pharmacological effects and differences in mechanisms of action

Apart from CGA, many other CGAs belonging to the CGAs family have garnered considerable attention due to their diverse biological activities and potential health benefits (Chang, 2022; Joneidi et al., 2024). The presence of many isomers is determined by the location and quantity of caffeoyl groups bonded to the quinic acid, which makes up the chemical structure of CGAs. These isomers not only differ in structure but also exhibit unique pharmacological activities. This section primarily focuses on the botanical drugs NCGA, CCGA, ICGA-A, ICGA-B, and ICGA-C. These substances have a wide range of medicinal qualities, including anxiolytic, hypolipidemic, antihypertensive, antidepressant, antibacterial, anti-inflammatory, anticancer, and antidiabetic effects (Hung et al., 2023; Bai et al., 2024; Song et al., 2024a; Tsai et al., 2024; Xu et al., 2024). These diverse biological activities highlight the significant potential of CGAs for application in drug development and functional food development.

### 6.1 Neochlorogenic acid

**FIGURE 3:**
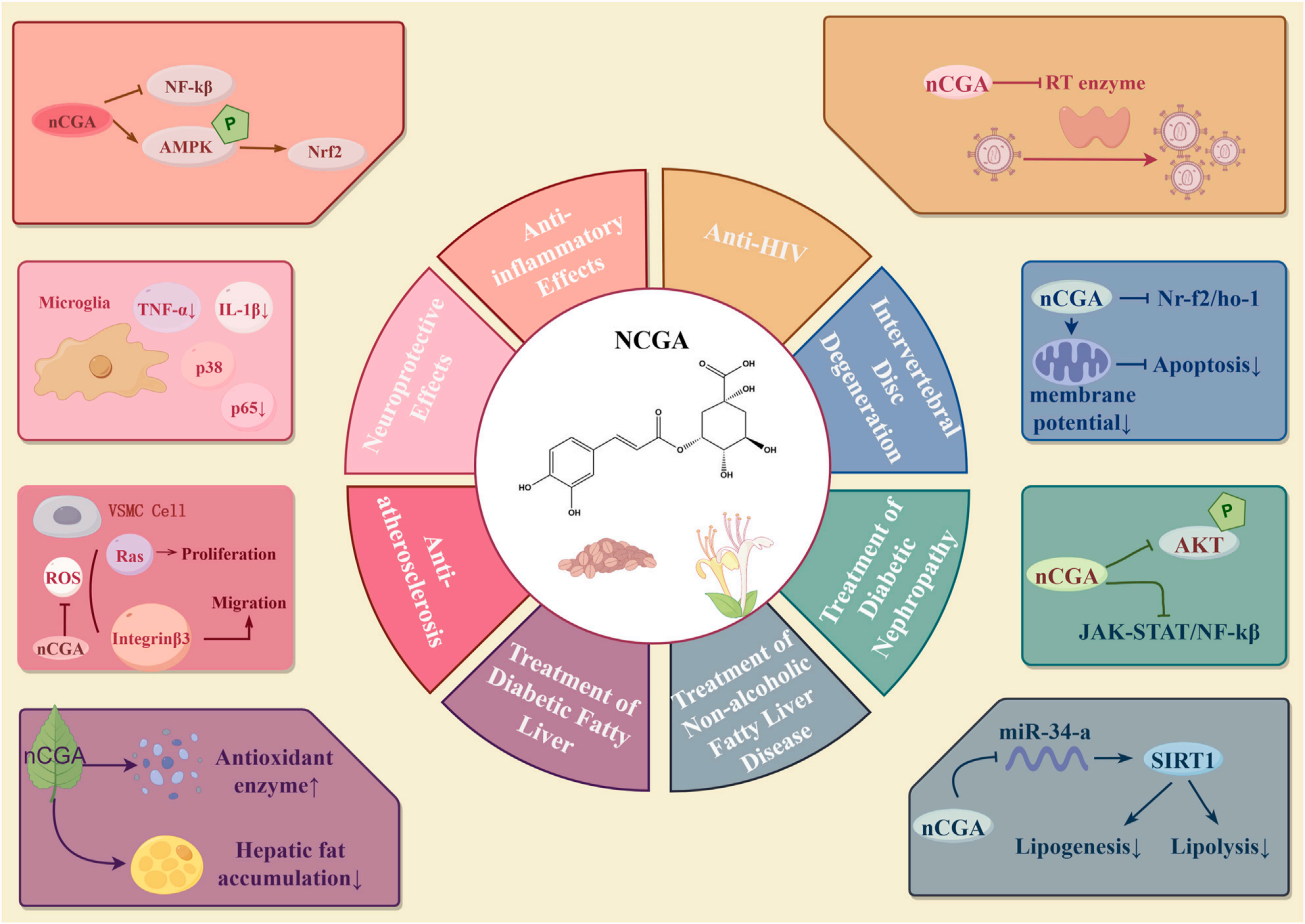
Pharmacological effects of neochlorogenic acid.

Neochlorogenic acid (NCGA), also known as 3-O-caffeoylquinic acid, is an isomer of CGA and exhibits multiple pharmacological activities. NCGA functions as an activator of the AMPK/Nrf2 signaling pathway, thereby inhibiting excessive macrophage-driven responses implicated in both acute and chronic inflammatory conditions (Park et al., 2018). It exerts neuroprotective effects by inhibiting pro-inflammatory pathways in activated microglia (Kim et al., 2015). Additionally, NCGA reduces the motility and expansion of vascular smooth muscle cells (VSMCs) to treat atherosclerosis by inhibiting focal adhesion kinase (FAK)/Rho GTPase regulator 3 (Rho GT3), PI3K/Akt, and Ras-related signaling pathways (Yang et al., 2022). NCGA controls lipid metabolism in a variety of ways. NCGA effectively increases the activity of hepatic antioxidant enzymes, which inhibits lipogenesis and prevents diabetes fatty liver, according to an experimental investigation (Tsai et al., 2024). According to another study, NCGA improves lipid and glucose metabolism by reducing insulin resistance brought on by inflammation and oxidative stress. In rats that have been given a high-fat, high-fructose diet (HFFD), it also increases insulin signaling while decreasing the expression of gluconeogenic and lipogenic genes. The management of non-alcoholic fatty liver disease is aided by these mechanisms taken together (Singdam et al., 2023). Furthermore, Yu et al. (2021b) discovered that NCGA reduces fat accumulation by downregulating miR-34a, which activates the SIRT1/AMPK pathway. Additionally, by enhancing body weight, insulin sensitivity, lipid profile, and renal function as well as by controlling the JAK-STAT, pAKT, Ras, and NF-κB signaling pathways, NCGA cures diabetic nephropathy (Hung et al., 2023). Additionally, experiments have shown that NCGA inhibits HIV-1 reverse transcriptase (RTase), indicating its potential for anti-AIDS (HIV) treatment (Li et al., 2021a). By modifying the Nrf2/HO-1 pathway, NCGA reduces oxidative stress in bone marrow mesenchymal stem cells (BMSCs), potentially providing a treatment approach for intervertebral disc degeneration. The Nrf2 pathway is probably implicated in the protective mechanism of NCGA against apoptosis, as it prevents the decline in mitochondrial membrane potential and guards against H_2_O_2_-induced apoptosis (Fang et al., 2024). In conclusion, through a variety of pathways, NCGA contributes significantly to inflammation, metabolic disorders, viral infections, and degenerative illnesses. Figure 3 provides a summary of NCGA’s pharmacological activities.

### 6.2 Cryptochlorogenic acid

**FIGURE 4:**
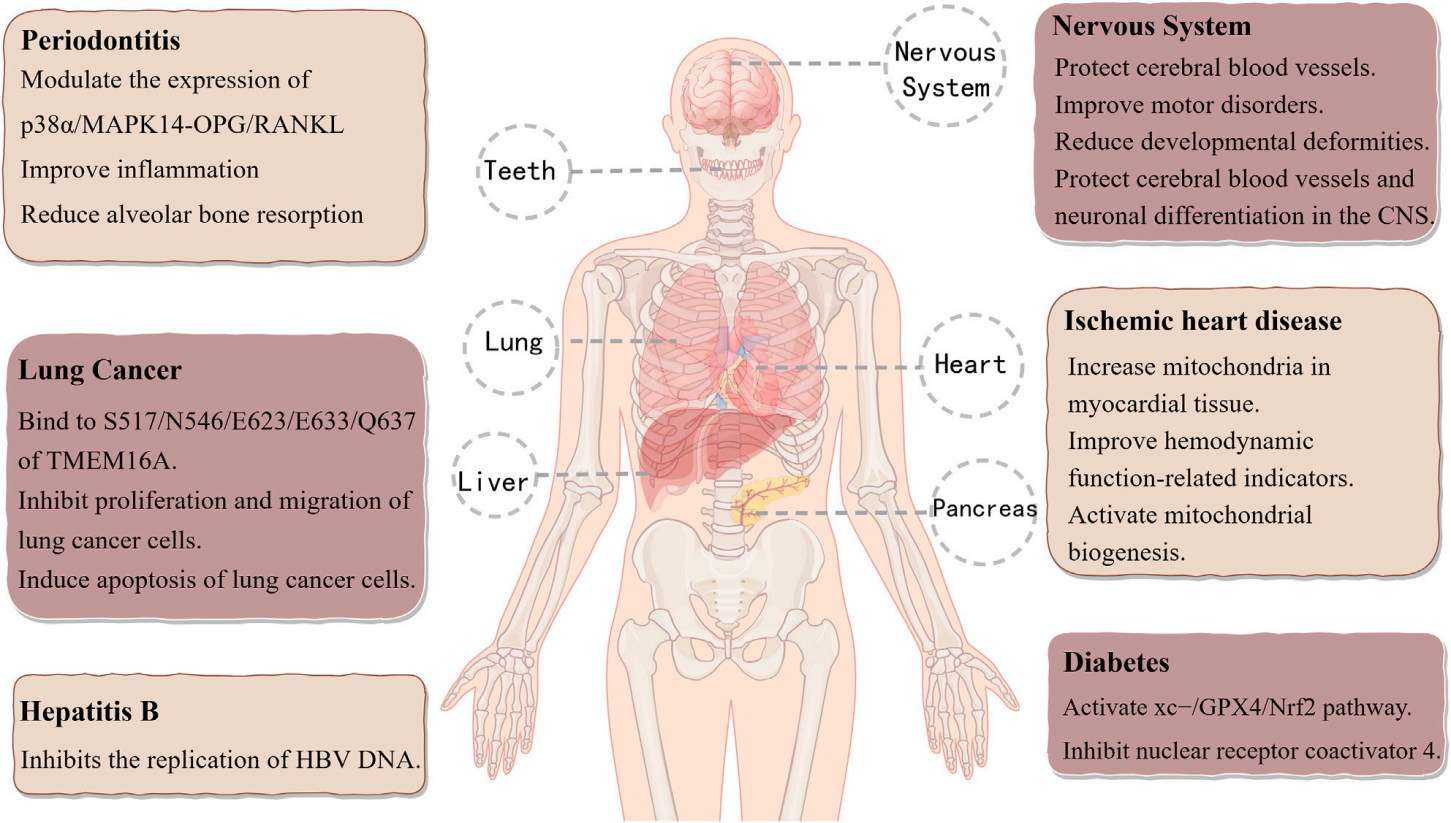
Therapeutic applications and mechanisms of action of cryptochlorogenic Acid.

Cryptochlorogenic acid (CCGA), also known as 4-caffeoylquinic acid, exhibits significant anti-inflammatory, hypoglycemic, and lipid-lowering activities (Ma et al., 2022; Vázquez-Atanacio et al., 2025). Song et al. (2024b) evaluated 11 components of Epimedium and discovered that CGA, NCGA, and CCGA significantly enhanced calcium deposition and reduced the number of multinucleated osteoclasts. Additionally, matrix metalloproteinase 9 (MMP-9) expression was markedly suppressed by CCGA. According to a recent study, CCGA increases the expression of the anti-inflammatory cytokine IL-10 while downregulating the production of pro-inflammatory cytokines like IL-6, IL-1β, and TNF-α. By modulating the RANKL/OPG signaling pathway, CCGA effectively reduces alveolar bone resorption, thus providing a protective effect on bone tissue. The anti-inflammatory and bone-protective properties of CCGA are mediated through antioxidant mechanisms to fight periodontitis and are probably linked to the suppression of the p38MAPK signaling pathway (Wang et al., 2021b). CCGA decreases LPS-induced oxidative stress through Nrf2 nuclear translocation and reduces LPS-induced inflammatory symptoms by modifying the NF-κB/MAPK signaling pathway (Zhao et al., 2020). Ji et al. (2021) utilized a zebrafish model to show that CCGA mitigates lead-induced neurotoxicity by inhibiting apoptosis and protecting cerebral blood vessels. This action increases the length of dopaminergic (DA) neurons, safeguards the cerebrovascular system and neuronal differentiation in the central nervous system (CNS), improves motor dysfunction, and regulates neurodevelopmental genes, as well as genes related to Parkinson’s disease and autophagy. These findings suggest that CGA, NCGA, and CCGA may serve as promising therapeutic agents for Pb-induced neurotoxicity, with CCGA exhibiting the most potent detoxification activity. Tong et al. (2024) used a Langendorff-perfused mouse heart ischemia/reperfusion (I/R) injury model to study cardiovascular diseases. They found that CCGA improves cell morphology, improves hemodynamic function, and increases mitochondrial density in I/R myocardial tissue. The results indicate that CCGA may be a potential treatment option for ischemic heart disease (IHD) and that it may be somewhat more effective than CGA and NCGA. By decreasing nuclear receptor coactivator 4 (NCOA4) in diabetes and triggering the cystine/glutamate antiporter system (XC^−^)/GPX4/Nrf2 pathway, CCGA prevents ferroptosis (Zhou, 2020). Furthermore, by attaching itself to TMEM16A, a target unique to lung cancer, CCGA efficiently prevents the development and metastasis of lung cancer cells (Bai et al., 2024). Additionally, with an IC_50_ value of 5.5 ± 0.9 μM, CCGA exhibits strong efficacy against HBV DNA replication (Zhao et al., 2014). Figure 4 shows the modes of action and therapeutic uses of CCGA.

### 6.3 Isochlorogenic acids

**FIGURE 5:**
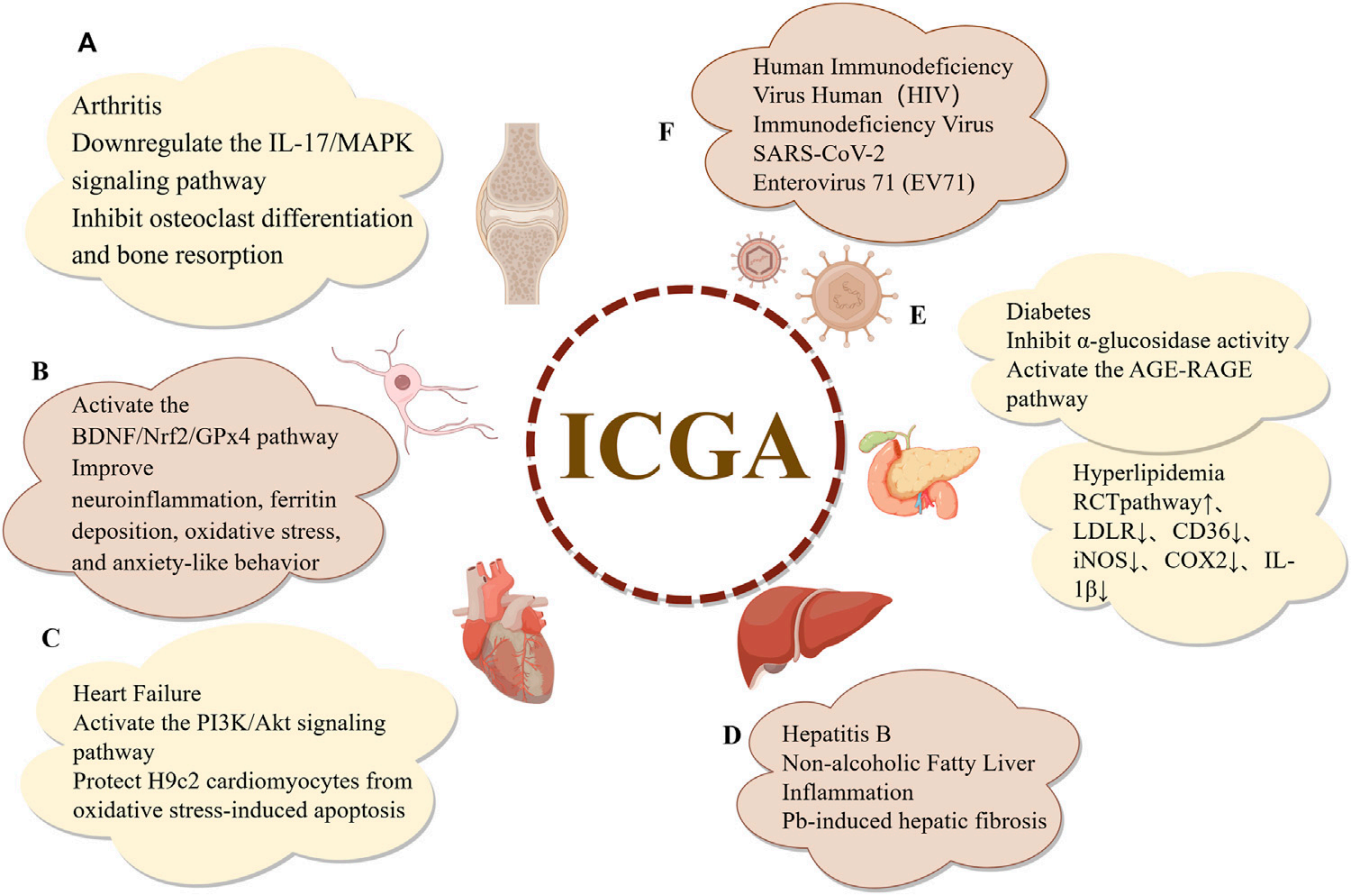
Pharmacological effects of isochlorogenic Acids. **(A)** Anti-inflammatory and antioxidant effects **(B)** Neuroprotective effects **(C)** Cardiovascular protective effects **(D)** Hepatoprotective effects **(E)** Effects on metabolic syndrome **(F)** Antiviral effects.

Isochlorogenic acids A, B, and C are dicaffeoylquinic acid derivatives formed through the esterification of quinic acid with different quantities of caffeic acids. These natural organic acids are isomers and are widely distributed throughout the plant kingdom. Numerous studies on the pharmacological properties of dicaffeoylquinic acids have been carried out over the last 20 years, demonstrating their important biological properties, which include lipid-regulating, anti-inflammatory, antiviral, antifibrotic, smooth muscle relaxation, antioxidant, and anti-atherosclerotic effects. The pharmacological activities of isochlorogenic acids are depicted in Figure 5. Owing to their structural differences, the pharmacological activities of these isomers also exhibit variations, which will be discussed in detail in the subsequent sections.

#### 6.3.1 Isochlorogenic acid A

Isochlorogenic acid A (ICGA-A), also known as 3,5-dicaffeoylquinic acid, is a natural phenolic acid (Behne et al., 2023). Over the past few years, as research has advanced, its potential applications in antioxidant, anti-inflammatory, hepatoprotective, and antiviral activities have been increasingly elucidated. Studies have demonstrated that ICGA-A can effectively scavenge free radicals, inhibit inflammatory responses, and exert antiviral effects against various viruses, thereby highlighting its significant potential in drug development and functional food applications. In research on rheumatoid arthritis and osteoporosis, Yang et al. (2023) reported that ICGA-A, an active component of Tubson-2 decoction, downregulates the IL-17/MAPK pathway and inhibits osteoclast differentiation and bone resorption *in vitro* in a dose-dependent manner. Additionally, Guo et al. (2024a) also found that ICGA-A inhibits brain-derived neurotrophic factor (BDNF), cAMP response element-binding protein (CREB), and Nrf2 by activating the BDNF/Nrf2/GPx4 pathway. This activity ameliorates oxidative stress, ferritin accumulation, neuroinflammation, and anxiety-like behaviors caused by lead (Pb). In terms of cardiovascular protection, ICGA-A protects H9c2 cardiomyocytes from oxidative stress-induced apoptosis by activating the PI3K/Akt signaling pathway (Bi et al., 2018). Regarding hepatoprotection, ICGA-A mitigates CCl_4_-induced hepatic fibrosis by inhibiting the high-mobility group box 1 (HMGB1)/TLR4/NF-κB signaling pathway (Liu et al., 2020b). Similarly, ICGA-A ameliorates Pb-induced hepatic inflammation and fibrosis by inhibiting the AMPK/MAPKs/NF-κB and STAT3/transforming growth factor β1 (TGF-β1)/Smad2/3 pathways (Guo et al., 2024b). In diabetes treatment, ICGA-A enhances glucose uptake by inhibiting α-glucosidase activity in HepG2 cells, thereby demonstrating potential therapeutic efficacy (Chen et al., 2020). Moreover, ICGA-A exhibits antiviral activity by inhibiting HIV-1 integrase (IN) and blocking the replication of HIV-1 virus in tissues (Robinson et al., 1996). Collectively, ICGA-A holds broad application prospects across multiple biomedical fields, and its diverse biological activities provide a robust scientific foundation for its future applications in drug development and functional foods.

#### 6.3.2 Isochlorogenic acid B

Isochlorogenic acid B (ICGA-B), or 4,5-dicaffeoylquinic acid, exhibits anti-inflammatory, neuroprotective, and hepatoprotective properties. Regarding anti-inflammatory MDA levels and the enhancement of antioxidant enzyme activity. Additionally, ICGA-B reduces Pb-induced brain inflammation by lowering levels of IL-6 and TNF-α. Phosphatidylinositol 3-kinase/protein kinase B (PI3K/AKT) and cAMP response element-binding protein (CREB) are phosphorylated by ICGA-B, which also increases the expression of BDNF. In particular, TLR4, MyD88, glycogen synthase kinase-3β (GSK-3β), and p38 are all decreased by ICGA-B. By altering the BDNF signaling pathway, ICGA-B often alleviates Pb-induced anxiety, depression, neuroinflammation, and oxidative stress (Shi et al., 2023b). Through the miR-122/hypoxia-inducible factor-1α (HIF-1α) signaling pathway, ICGA-B inhibits a number of profibrotic factors and significantly prevents fibrosis in non-alcoholic steatohepatitis (NASH) (Liu et al., 2019). For the treatment of NASH, this discovery offers fresh perspectives and possible therapeutic targets.

#### 6.3.3 Isochlorogenic acid C

Isochlorogenic acid C (ICGA-C), chemically known as 3,4-dicaffeoylquinic acid, has been shown in a recent study to inhibit the proliferation of human fibroblast-like synoviocytes and promote apoptosis. ICGA-C also inhibits the nuclear translocation of NF-κB, ERK, and JNK. In collagen-induced arthritis (CIA), ICGA-C has been shown to considerably reduce edema in the hind paws, lower arthritis scores, lessen bone tissue destruction, and ease synovitis. Through the Erk/JNK/NF-κB pathway, ICGA-C may reduce inflammation-induced synovial proliferation by promoting apoptosis and preventing excessive activation of inflammatory cells (Liu et al., 2023a). By inhibiting the expression of genes involved in cholesterol uptake, such as LDLR and CD36, and promoting the LXRα-mediated reverse cholesterol transport (RCT) pathway, ICGA-C prevents the development of macrophage foam cells. Furthermore, it lowers the synthesis of inflammatory mediators such as IL-1β, COX2, and iNOS. Without affecting hepatic LXRα, ICGA-C can speed up RCT, causing a hypolipidemic effect without causing hepatic steatosis. In order to treat hyperlipidemia without resulting in hepatic steatosis, ICGA-C may be used as a novel RCT promoter (Zheng et al., 2022). By altering glutathione redox equilibrium, ICGA-C can stop enterovirus 71 (EV71) infection (Cao et al., 2017). Furthermore, research has demonstrated that ICGA-A and ICGA-C have angiotensin-converting enzyme (ACE)-inhibitory activity, indicating that isochlorogenic acids may have antihypertensive effects (Oh et al., 2002).

Several studies have demonstrated that isochlorogenic acids A, B, and C exhibit potential for inhibiting SARS-CoV-2 infection (Wu et al., 2023). In addition, CGA, CCGA, NCGA, ICGA-A, ICGA-B, and ICGA-C effectively inhibit the replication of hepatitis B virus (HBV) DNA. Furthermore, ICGA-A, ICGA-B, and ICGA-C also inhibit the secretion of hepatitis B surface antigen (HBsAg) and hepatitis B e antigen (HBeAg) (Zhao et al., 2014).

Although CCGA, NCGA, and ICGA have shown a variety of pharmacological activity, including lipid-lowering, hypoglycemic, anti-inflammatory, and antioxidant properties, the exact mechanisms behind their therapeutic benefits remain unclear. Current research is predominantly based on *in vitro* experiments, whereas *in vivo* studies are comparatively scarce, which hampers a comprehensive understanding of these activities. To advance our understanding of these botanical drugs, future research should prioritize comprehensive *in vivo* studies and detailed mechanistic investigations. Such efforts are critical for fully characterizing their pharmacological properties and exploring their potential clinical uses.

## 7 Comparative analysis of the six members of the chlorogenic acids family

CGAs have been demonstrated to possess multiple pharmacological activities, such as anti-inflammatory, antioxidant, and metabolic regulation, which are primarily mediated by core signaling pathways, including PI3K/Akt, NF-κB, Nrf2, and AMPK. However, as can be seen from the previous text and Table 5, the current research exhibits significant imbalances. CGA, as the most extensively studied member, has been demonstrated to have broad-spectrum pharmacological functions, including anti-inflammatory and antioxidant activities, neuroprotection, cardiovascular protection, hepatoprotection, and antiviral effects. NCGA and CCGA are the next most studied, while ICGA-B and ICGA-C, limited by complex extraction and separation techniques, still have significant gaps in research regarding their biological activity profiles and mechanisms of action. It is worth noting that although CGA, NCGA, CCGA, and ICGA-A all have anti-inflammatory and antioxidant activities, they exhibit specific differences in their targets—CGA can treat multiple diseases through anti-inflammatory and antioxidant effects (Table 3), NCGA mainly focuses on protection against skin photoaging, CCGA focuses on the treatment of periodontitis, and ICGA-A improves arthritis symptoms. We found that, although all six derivatives have shown potential for diabetes treatment (Table 6), the mechanisms of action of CGA, NCGA, CCGA, ICGA-A, and ICGA-B exhibit significant heterogeneity, suggesting that different structural derivatives may achieve similar pharmacological effects through unique molecular pathways.

**TABLE 5:** Comparative analysis of six CGAs: Structural features, biological targets, bioactivities, and pharmacokinetic propertie.

Name	Structural features	Main biological targets	Core biological activities	Pharmacokinetic properties
CGA	5-O-Caffeoylquinic acid	Pathways: SIRT1/PGC-1α/BACE1, mTOR/TFEB, Akt/Erk1/2, AMPK/PGC-1α, HIF-1α/AKT, LncNeat1/NLRP3, TLR4/NF-κB, IL-13/miR-21/Smad7, PGC-1α/UCP-1, PI3K/Akt/mTORC, RTK/MEK/ERK Molecules: TNF-α↓, IL-1β↓, IL-6↓, IL-8↓, PGE2↓, Nrf2↑, SIRT1↑, CA↓, NO↑, CD14↓, TFF-3↓, NF-κB↓, ALKBH5↓, DNMT1↓, MMP-2↓, MMP-9↓, Bcl2↓, Bcl-xL↓, p53↑, caspase-3↑, RTK↓, VEGF↓, EGF↓, CD34↓, IL-10↓, TGF-β↓, BMI1↓, SOX2↓, Bcr-Abl↓, Notch1v↓, STAT3↓, HIF-1α↓	Anti-inflammatory and antioxidant, antibacterial, antiviral, neuroprotective, cardioprotective, nephroprotective, gastrointestinal protective, hepatoprotective, improving metabolic syndrome, antitumor activity, anti-osteoporosis, leukemia treatment, improving PCOS	Phase I clinical study Dose: 3 mg/kg Cmax: 2,961 ng/mL Tmax: 1.0 h t1/2: 1.27 h AUC 0- ∞ : 7,814 h*ng/mL (Kang et al., 2023)
NCGA	3-O-Caffeoylquinic acid	Pathways: AMPK/Nrf2, XC-/GPX4/Nrf2, PI3K/Akt, SIRT1/AMPK, JAK-STAT, Nrf2/HO-1, NF-κB Molecules: FAK↓, small GTP proteins↓, Ras↓, miR-34a↓, pAKT, RT enzyme↓, Nrf2↓	Anti-inflammatory and antioxidant (anti-photoaging), neuroprotective, cardioprotective (atherosclerosis), hepatoprotective (diabetic fatty liver), improving metabolic syndrome (diabetes, non-alcoholic fatty liver, diabetic nephropathy), antitumor activity (cervical cancer), antiviral (anti-HIV), improving intervertebral disc degeneration	N/A
CCGA	4-O-Caffeoylquinic acid	Pathways: RANKL/OPG, NF-κB/MAPK, Nrf2 Molecules: MMP-9↓, IL-6↓, IL-1β↓, TNF-α↓, IL-10↑, NCOA4↓, FAK↓	Anti-inflammatory and antioxidant (periodontitis), neuroprotective, cardioprotective (iron-deficiency cardiomyopathy), hepatoprotective, improving metabolic syndrome (diabetes), antitumor activity (lung cancer), antiviral (against H9N2-AIV virus)	N/A
ICGA-A	3,5-O-Dicaffeoylquinic acid	Pathways: IL-17/MAPK, BDNF/Nrf2/GPx4, PI3K/Akt, HMGB1/TLR4/NF-κB, AMPK/MAPKs/NF-κB, STAT3/TGF-β1/Smad2/3 Molecules: BDNF↓, CREB↓, Nrf2↓, IN↓	Anti-inflammatory and antioxidant (arthritis), neuroprotective, cardioprotective (heart failure, blood pressure reduction), hepatoprotective (non-alcoholic fatty liver, acute liver injury), improving metabolic syndrome (diabetes), antiviral (CDV, HIV, SARS-CoV-2, EV71), nitration inhibition	N/A
ICGA-B	4,5-O-Dicaffeoylquinic acid	Pathways: PI3K/AKT, miR-122/HIF-1α Molecules: MDA↓, TNF-α↓, IL-6↓, BDNF↑, CREB↑	Neuroprotective, hepatoprotective (acute liver injury, non-alcoholic steatohepatitis), improving metabolic syndrome (diabetes), antiviral	N/A
ICGA-C	3,4-O-Dicaffeoylquinic acid	Pathways: NF-κB, Erk/JNK/NF-κB Molecules: Erk↓, JNK↓, RCT↑, LDLR↓, CD36↓, iNOS↓, COX2↓, IL-1β↓	Improving metabolic syndrome (hyperlipidemia), antiviral, cardioprotective (blood pressure reduction)	N/A

**TABLE 6:** Comparison of mechanisms of antidiabetic action of CGA, NCGA, CCGA, and ICGA-A.

Name	Types	Model	Dosing and treatment duration	Primary mechanism of action	References
CGA	*in vitro*	3T3-L1 cell	50 mM for 24 h	Activates GPR40, activates the IP3 receptor pathway, increases [Ca2+]i, thereby promoting insulin secretion.	Sanchez et al. (2017)
NCGA	*in vivo*	Male Sprague-Dawley rats	50 mg/kg for 6 weeks	Reduces insulin resistance and expression of gluconeogenesis genes (PEPCK and G6Pase), enhances insulin signaling.	Singdam et al. (2023)
CCGA	*in vivo* *in vitro*	animal: Sixty Sprague-Dawley rats cell: INS-1 cells	animal: 15 mg/kg, 30 mg/kg, 60 mg/kg for 2 weeks cell: 10 μM, 25 μM, 50 μM for 24 h	Activates the cystine/glutamate transporter system/GPX4/Nrf2 to inhibit ferroptosis, exerting anti-diabetic effects.	Zhou (2020)
ICGA-A	*in vitro*	HepG2 cell	0–12 μM for 24 h	Inhibits α-glucosidase activity, increases insulin secretion and promotes pancreatic tissue repair.	Chen et al. (2020)

Although the pharmacological activity studies of the chlorogenic acids family members have revealed their multi-target action characteristics, their pharmacokinetic research framework still has significant deficiencies, which directly impact the evaluation of clinical translation potential. Currently, only the team of Chunhua Liu has indirectly obtained the fundamental metabolic parameters of the six derivatives through herbal botanical drug: all botanical drugs have a T_max_ of approximately 0.25 h in mice, indicating rapid absorption characteristics; however, the elimination half-life (T_1/2_) shows significant differences—CGA is the longest (3.97 h), NCGA, CCGA, ICGA-A, and ICGA-B range from 3.0 to 3.6h, while ICGA-C is the shortest (2.88 h) (Liu et al., 2022). These data suggest that structural differences among the derivatives may significantly affect metabolic stability. Existing studies depend on compound environments to obtain data, lack independent pharmacokinetic studies on NCGA, CCGA, and each subtype of ICGA, and cannot rule out the interference of component interactions, which significantly hinder dose optimization and dosage form design. Therefore, in the future, systematic pharmacokinetic characteristic analysis of NCGA, CCGA, and ICGA should be carried out to provide a theoretical basis for optimizing dosing regimens.

## 8 Stability of CGAs in dietary systems and interactions with other components

The diverse pharmacological activities of CGAs, demonstrated across multiple levels and targets in cellular and animal models, have established a robust theoretical basis for their potential as therapeutic agents. However, as a predominant polyphenolic component in daily diets, the health benefits of CGAs are not realized in isolation. Instead, they are influenced by interactions within complex food matrices. These interactions significantly impact the bioavailability, metabolic pathways, and physiological functions of CGAs, bridging the gap between their *in vitro* activities and *in vivo* effects.

### 8.1 Effects of dietary intake and processing on CGAs

Content Coffee, fruits, and vegetables are primary dietary sources of CGAs. Research indicates that coffee is the main contributor to daily CGA intake, with a single 200 mL cup containing approximately 50–150 mg of CGAs. Frequent coffee consumers may ingest 0.5–1.0 g per day, a level far exceeding that provided by medicinal plants or traditional Chinese medicine herbs (Scalbert and Williamson, 2000; Clifford et al., 2017). When evaluating the health effects of CGAs, it is crucial to consider their dietary context and the changes that occur during processing.

Food processing significantly affects the stability and final content of CGAs. Processes such as roasting, heating, and fermentation can lead to hydrolysis, degradation, or structural transformation of CGAs (Badmos and Kuhnert, 2025). For example, deep roasting of coffee beans can result in a loss of over 50% of CGAs, with partial conversion to chlorogenic acid lactones. Similarly, fruits and vegetables experience a substantial reduction in CGAs after blanching or boiling due to leaching effects (Budryn et al., 2009; Kamiyama et al., 2015). Fermented foods alter the composition and content of CGAs through microbial enzymatic hydrolysis (Kalinowska et al., 2023).

### 8.2 Stability challenges and preservation strategies for CGAs in food processing

The stability of CGAs is challenged by their sensitivity to heat, pH, and light, primarily due to their ester bonds and phenolic hydroxyl groups. High temperatures can trigger hydrolysis, isomerization, and oxidation reactions, causing a loss of over 50% of CGAs in deeply roasted coffee beans (Budryn et al., 2009). CGAs are also highly susceptible to degradation in alkaline conditions (pH > 6.0), while they remain relatively stable in acidic environments (pH < 3.0) (Wang et al., 2021c). To enhance CGA stability during food processing and storage, several strategies have been developed. Microencapsulation using maltodextrin and gum arabic as wall materials effectively isolates CGAs from light, oxygen, and heat, significantly improving their retention rate (Ibrahim et al., 2025). Adding antioxidants and metal chelators to food systems, along with maintaining an acidic environment, is another straightforward and effective stabilization method. Non-thermal processing technologies, such as high-pressure processing (HPP) and pulsed electric field (PEF), offer alternatives to traditional thermal processing, reducing CGA degradation. It is important to note that current research on CGA stability predominantly focuses on 5-CQA. Knowledge of the degradation kinetics and stabilization strategies for other derivatives, such as NCGA and CCGA, remains limited. Future research should aim to fill these gaps to support the precise application of CGAs in both food and pharmaceutical industries.

### 8.3 Interactions of CGAs with other dietary components

#### 8.3.1 Polyphenolic components

CGAs can interact synergistically with other polyphenolic compounds to enhance their bioactivity. For example, combining tea catechins with CGAs boosts antioxidant effects. When CGAs form complexes with egg white proteins, they also improve emulsion stability (Sun et al., 2020). Anthocyanins, natural pigments with poor color stability, can benefit from CGAs, which significantly enhance their color intensity and stability through hydrogen bonding and π-π stacking interactions (Singh et al., 2025) Kang et al. (Kang and Koh, 2023) found that adding CGAs increased the retention rate and hyperchromic effect of anthocyanins in chokeberries. Additionally, animal experiments by Ariane Rocha Bartolomeu revealed that caffeine and CGA synergistically inhibit early carcinogenesis in chemically induced colon cancer models, likely by regulating proliferation, apoptosis, and inflammatory pathways (Bartolomeu et al., 2022).

#### 8.3.2 Proteins

The interaction between proteins and CGAs has garnered significant attention recently, both for functional modification and the development of new bioactive particles. Xu et al. (Xu et al., 2019) discovered that CGAs reduce the allergenicity of whey protein (WPI) while improving its solubility, emulsifying ability, foaming capacity, and antioxidant potential. Alongi et al. (Alongi et al., 2019) reported that combining CGAs with milk and high-pressure homogenization (HPH) treatment increased their bioavailability from 25% to over 50%, promoting micelle formation and reducing degradation during digestion. Adding fat (0.1%–7.1%) further enhanced CGA stability by promoting micelle formation and protein complexation.

These interactions are influenced by several factors, including the structure and charge of proteins, CGA concentration, binding site characteristics, and environmental pH and temperature. The consequences of these interactions can alter both the functional properties of proteins, such as thermal stability and emulsifying ability, and their nutritional attributes, including antioxidant activity and digestibility (Tarahi et al., 2024).

#### 8.3.3 Dietary fiber

The interaction between soluble dietary fiber and CGAs can enhance the bioefficacy of CGAs. Bozheng Li et al. (Li et al., 2024) used isothermal adsorption technology to create a complex of auricularia auricula-judae soluble dietary fiber (ASDF) and CGA (ASDF-CGA). This complex had a higher molecular weight and enhanced thermal stability compared to ASDF alone. Morphological analysis revealed smaller particle size, increased specific surface area, and a more porous, uniform structure. *In vitro* experiments confirmed that ASDF-CGA had higher cholesterol and bile acid adsorption capacity, indicating enhanced hypolipidemic activity. This complex also significantly increased CGA bioavailability. Wei Ji et al. (Ji et al., 2023) demonstrated that CGAs could structurally modify pea dietary fiber, improving its adsorption performance and potential health effects. These findings suggest that non-covalent complexation of dietary fiber and polyphenols is an effective strategy to enhance bioactivity by improving stability and bioavailability.

## 9 Summary and outlook

CGAs, as multipotent phytochemicals, have been proven to possess various therapeutic potentials, such as cardiovascular protection, regulation of blood glucose and lipids, antibacterial and antiviral effects, and anticancer properties. These attributes show broad prospects in the fields of pharmaceuticals, nutraceuticals, and functional foods. This review systematically combs through the existing literature to further clarify the differences in the activities of different CGAs components in antioxidant, anti-inflammatory, and targeted therapy. It also preliminarily reveals the potential correlation between their structure and function, providing a theoretical basis for subsequent targeted development.

The main advantage of this review lies in the extensive integration and comparison of the pharmacological activities and mechanisms of multiple components in the chlorogenic acids family. It highlights the functional characteristics of different CGA derivatives and the current research gaps. There are still several key issues in this field. First, the long-term safety and pharmacokinetic behavior of CGAs at high doses are not clear and need systematic evaluation. Second, CGAs in traditional Chinese medicine injections may cause allergic reactions, and a more precise safety evaluation system needs to be established. Third, the evidence for clinical translation is weak. There are interspecies metabolic differences in existing animal models. Future work should strengthen clinical and evidence-based medical research. In terms of mechanisms, most existing studies focus on a single CGA component. Research on other family members such as NCGA, CCGA, and ICGA is relatively scarce. There is also a lack of systematic activity comparison between different CGAs. This is mainly due to the complex extraction and separation of chlorogenic acid components, which leads to insufficient exploration of the mechanisms of individual compounds. In turn, this restricts their comprehensive development and utilization.

In the future, efforts should be made to conduct horizontal comparative studies of important members such as CGA, NCGA, CCGA, and ICGA in anti-inflammatory, antioxidant, and targeted therapy. Combined with advanced technologies such as computer-aided drug design and multi-omics analysis, these studies will deeply explore the structure-activity relationships between key structural features such as hydroxyl substitution and ester bond configuration and biological activity. This will promote the translation of CGAs from basic research to clinical and application transformation. Strengthening the research on the bioavailability, homeostasis regulation, and actual health effects of dietary-derived CGAs will promote the transformation of CGAs from basic scientific research to clinical nutrition and precision food.
